# Cryo2RT: a high-throughput method for room-temperature macromolecular crystallography from cryo-cooled crystals

**DOI:** 10.1107/S2059798324006697

**Published:** 2024-07-25

**Authors:** Chia-Ying Huang, Sylvain Aumonier, Vincent Olieric, Meitian Wang

**Affiliations:** a Swiss Light Source, Center for Photon Science Paul Scherrer Institute Forschungsstrasse 111 5232Villigen PSI Switzerland; University of Cambridge, United Kingdom

**Keywords:** macromolecular crystallography, room temperature, Cryo2RT, ligand binding, synchrotron beamlines

## Abstract

Advances in structural biology have relied heavily on synchrotron cryo-crystallography and cryogenic electron microscopy to elucidate biological processes and for drug discovery. However, disparities between cryogenic and room-temperature (RT) crystal structures pose challenges. Here, Cryo2RT, a high-throughput RT data-collection method from cryo-cooled crystals that leverages the cryo-crystallography workflow, is introduced. Tested on endothiapepsin crystals with four soaked fragments, thaumatin and SARS-CoV-2 3CL^pro^, Cryo2RT reveals unique ligand-binding poses, offers a comparable throughput to cryo-crystallography and eases the exploration of structural dynamics at various temperatures.

## Abstract 2

A pragmatic method, Cryo2RT, to obtain high-throughput room-temperature structures from cryo-cooled crystals has been demonstrated and applied to ligand-binding studies.

## 1. Introduction

In structural biology, synchrotron cryo-crystallography and cryogenic electron microscopy stand as cornerstones for understanding the fundamental processes of life and developing novel medicines (Shoemaker & Ando, 2018 ▸). However, growing evidence shows that the conformational distributions of protein side chains, ligand-binding poses and the structure of ordered water molecules may differ significantly between cryogenic (cryo) and room-temperature (RT) crystals (Fraser *et al.*, 2009 ▸, 2011 ▸; Keedy *et al.*, 2015 ▸, 2018 ▸; Fischer *et al.*, 2015 ▸; Skaist Mehlman *et al.*, 2023 ▸). Recent advances in sample delivery (Martiel *et al.*, 2019 ▸; Pearson & Mehrabi, 2020 ▸), X-ray detectors (Förster *et al.*, 2019 ▸) and data-acquisition methods (Winter *et al.*, 2019 ▸) have enabled routine high-resolution X-ray structure determination at or near RT (Fischer, 2021 ▸; Thorne, 2023 ▸; Doukov *et al.*, 2020 ▸), which not only reveals structural heterogeneity but also allows the investigation of dynamic structural changes, both of which are essential for biological functions (Ando, 2023 ▸).

**Figure 1:**
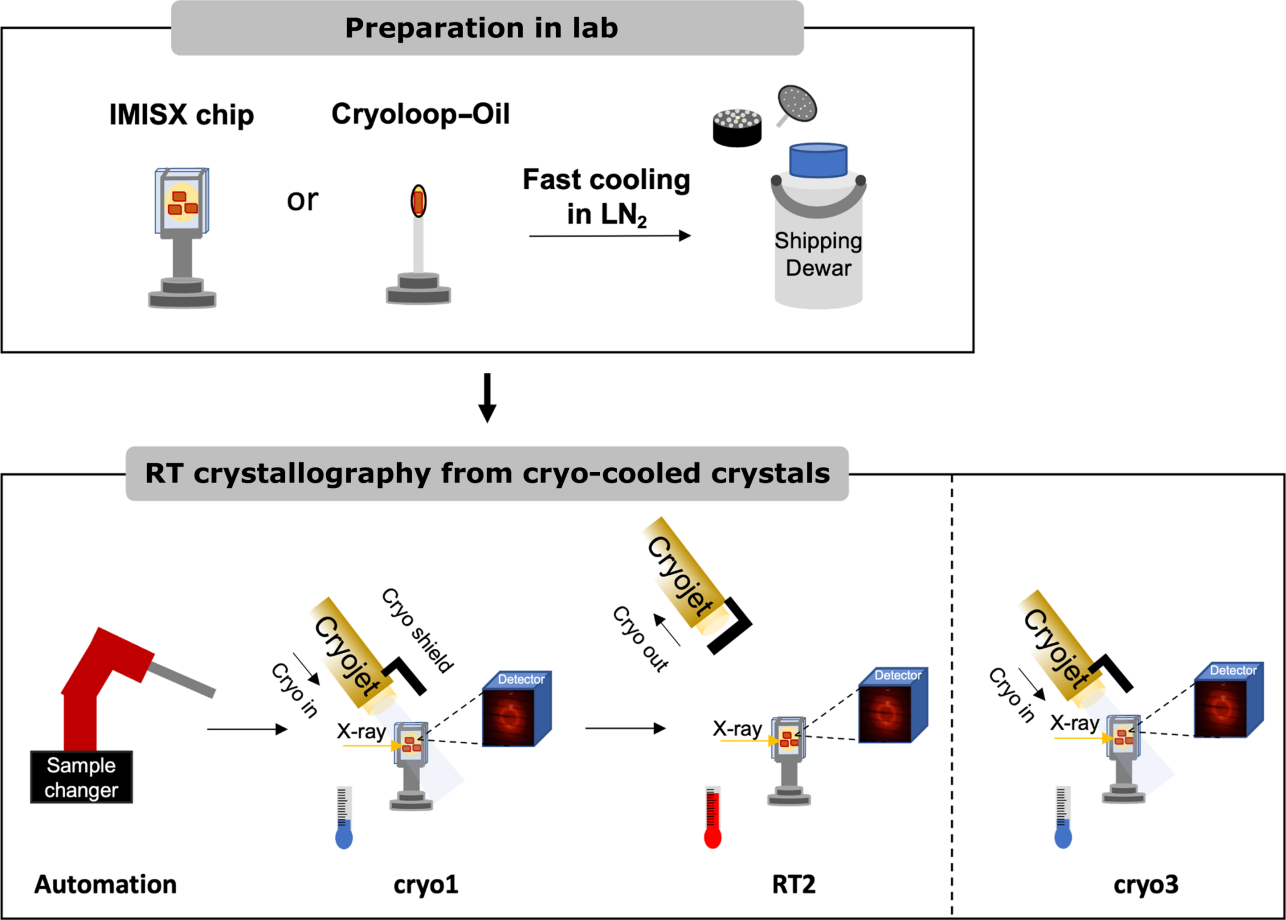
The Cryo2RT workflow. Samples are mounted on IMISX chips or cryoloops, snap-cooled and stored in liquid nitrogen (LN_2_) before shipping to the synchrotron. At the beamline, the samples are mounted on the goniometer using a sample changer under cryo conditions. RT data are collected after stopping the LN_2_ cryostream (RT2) after the first data set (cryo1) has been collected under cryo conditions at 100 K. Finally, the sample can be cryo-cooled again for another cryo data collection (cryo3) and stored back in LN_2_.

While the benefits of RT crystallography are appealing, one major barrier to its widespread adoption is the transportation of delicate protein crystals from research laboratories to synchrotron facilities (Thorne, 2023 ▸), together with the inevitable need for coordination between sample preparation and synchrotron beamtime to preserve crystal integrity. In contrast, cryo-crystallography (Garman & Schneider, 1997 ▸) has effectively addressed such issues, leading to the seminal success of synchrotron structural biology (Sweet, 1998 ▸; Duke & Johnson, 2010 ▸; Grabowski *et al.*, 2021 ▸). In this study, we introduce a pragmatic high-throughput RT data-collection method, Cryo2RT (Fig. 1 ▸), that takes advantage of the well established workflow for synchrotron cryo-crystallography. The procedure involves crystal cooling in liquid nitrogen (LN_2_) at the laboratory, shipping to the synchrotron under cryogenic conditions, as is commonly practiced, and thawing the crystals on the goniometer immediately before X-ray data collection.

Cryo-cooling and annealing were studied extensively in the early days of cryo-crystallography, with a focus on optimizing the snap-cooling process and minimizing the impact of cryo-cooling on crystal diffraction quality (Kriminski *et al.*, 2002 ▸; Juers *et al.*, 2007 ▸). Cooling/thawing cycles have also been exploited to trap protein intermediate states with temperature-controlled kinetic cryo-crystallography (Schlichting*et al.*, 2000 ▸; Bourgeois & Royant, 2005 ▸; Weik & Colletier, 2010 ▸). However, its general use for RT structure determination has not been fully exploited.

Here, we used endothiapepsin (EP) soaked with four different fragments (Supplementary Fig. S1), thaumatin (Thau) and SARS-CoV-2 3CL^pro^ (3CL^pro^) as test systems to show that RT structures can be obtained after the cooling/thawing process. For EP, we also captured unique ligand-binding poses that had not been seen in cryo structures. Our Cryo2RT method applies to conventional single-crystal data collection and achieves a comparable throughput to cryo-crystallography, enabling high-throughput fragment-based screening at RT: a highly sought-after method in structure-based drug discovery. Furthermore, the method is applicable to serial crystallo­graphy approaches, facilitating the exploration of functional binding modes and structural dynamics at multiple temperatures.

## 2. Materials and methods

### 2.1. Crystallization and fragment soaking

Endothiapepsin (EP) was obtained as Suparen 600 (Product No. 114604-516) from Prochem AG. The protein was exchanged into a buffer consisting of 100 m*M* sodium acetate pH 4.6 and then concentrated to a final concentration of 50 mg ml^−1^. The EP crystals were grown using two different methods. Medium-sized EP crystals (70 × 30 × 20 µm) for the IMISX chip were obtained using the batch crystallization method. 100 µl of the EP solution at 100 mg ml^−1^ was mixed with 200 µl of a crystallization solution consisting of 100 m*M* sodium acetate pH 4.6, 100 m*M* ammonium acetate, 30 m*M* MgCl_2_, 28%(*w*/*v*) PEG 4000. The resulting mixture was placed in a tube on a rotator operating at 20°C. These crystals were stored at 4°C until the experiment. On the other hand, single large-sized EP crystals (400 × 50 × 20 µm) for the cryoloop were grown using the vapor-diffusion method as described previously (Huang *et al.*, 2022 ▸). The crystallization solution consisted of 100 m*M* ammonium acetate, 100 m*M* sodium acetate pH 4.6, 26–30%(*v*/*v*) PEG 4000.

The fragments TL00150, AC40075, JFD03909 and AC39729 (Supplementary Table S1) were purchased from Sigma–Aldrich. Fragment soaking was performed at 23°C with a targeted DMSO concentration of 15%(*v*/*v*). The final fragment concentration in the crystal drop was 15 m*M*. The crystals were soaked for 3 h and harvested for cryogenic and RT X-ray experiments.

Thaumatin (Thau) was purchased from Sigma (catalogue No. T7638). The protein was prepared by dissolving 100 mg of thaumatin powder in 1 ml Milli-Q water to reach a concentration of 100 mg ml^−1^. The thaumatin crystals were obtained using the vapor-diffusion method by mixing 1 µl protein solution with 1 µl crystallization solution, and stabilized with 500 µl crystallization solution. The crystallization solution consisted of 1.6 *M* sodium potassium tartrate. After an incubation period of 16–18 h, thaumatin crystals grew to dimensions of 450 × 320 × 120 µm.

SARS-CoV-2 3CL^pro^ (3CL^pro^) was obtained as described previously (Huang *et al.*, 2024 ▸). Briefly, an 8 mg ml^−1^ protein solution was used and crystallization was performed using the vapor-diffusion method by mixing the protein and crystallization solutions in a 1:1 ratio. The crystallization solution consisted of 30 m*M* sodium nitrate, 30 m*M* disodium hydrogen phosphate, 30 m*M* ammonium sulfate, 100 m*M* MES–imidazole pH 6.5, 20%(*w*/*v*) PEG 550 MME, 10%(*w*/*v*) PEG 20K (Morpheus condition C1). The crystals grew to maximum dimensions (150 × 80 × 20 µm) in three days.

### 2.2. Crystal harvesting

Two different sample-delivery methods were investigated depending on the available crystal sample size. For the IMISX chip setup, a 1 µl sample was placed on an IMISX plate and a single well was cut out and mounted on a 3D-printed holder as described previously (Huang *et al.*, 2020 ▸). For the cryoloop single-crystal setup, crystals were mounted using MicroLoops E (SKU M8-L18SP-50V) and reusable B5 goniometer bases (SKU GB-B5-R-20) (MiTeGen, Ithaca, New York, USA). Subsequently, the crystals were wrapped in a drop of either Paratone-N oil (Hampton Research CryoPro HR2-132, tube 11) or paraffin oil (CryoPro HR2-132, tube 12). Both the IMISX chip and cryoloop were snap-cooled in LN_2_ and stored in UniPucks.

### 2.3. X-ray data collection

X-ray diffraction data were collected on beamlines PXI-X06SA and PXII-X10SA at the Swiss Light Source (SLS), Villigen PSI, Switzerland and BM07 (FIP-2) at the European Synchrotron Radiation Facility (ESRF). The temperature at the sample position was controlled using a cryostream (Cryostream 800, Oxford) with a nozzle-to-sample distance of 5 mm for the cryogenic experiments (100 K). The cryostream nozzle was shielded and retracted (to avoid blowing on the equipment) for data collection at room temperature (296 K) (Fig. 1 ▸).

The beamline sample changer was used to mount the samples (Martiel *et al.*, 2020 ▸), and X-ray data collections were carried out using the following procedure (Fig. 1 ▸). Three data sets were collected from each EP sample. The first one was at 100 K (cryo1); the cryostream was then shielded and retracted and the second data set was collected at 296 K (RT2) after the crystals had been stabilized from any motion induced by the thawing process in both the IMISX chips and the cryoloops. Finally, the cryostream was moved back in to cryo-cool the sample for the third data set at 100 K (cryo3), which was taken as a control. Only one RT data set and one cryo data set were collected for Thau and 3CL^pro^. We used the same or a similar X-ray dose for each RT and cryo data set. The X-ray doses (Supplementary Tables S2–S5), estimated based on equations (1) and (5) in Holton (2009 ▸), were kept below 500 kGy for each crystal, which is considered to be low for cryo-crystallography (Owen *et al.*, 2006 ▸) and is also below the radiation-damage safety margin for RT crystallography (Southworth-Davies *et al.*, 2007 ▸; Owen *et al.*, 2012 ▸; Warkentin *et al.*, 2012 ▸, 2017 ▸; Roedig *et al.*, 2016 ▸).

For the IMISX chip, we performed multi-crystal data acquisition by manually selecting and collecting a small wedge of data from each crystal (Huang *et al.*, 2015 ▸, 2016 ▸). The X-ray wavelength, collection speed, flux and beam size were 1 Å (12.398 keV), 40 deg s^−1^, 2.2–8.8 × 10^11^ photons s^−1^ and 75 × 40 µm, respectively. Each complete data set consists of several partial data sets of 30–110° each, depending on the crystal size. The measurements were carried out at PXII-X10SA using an EIGER2 16 M detector.

For cryoloop single-crystal data collection, we collected each data set from a different crystal position, minimizing radiation-damage contamination between the data sets. The measurements of EP cryoloop samples treated with Paratone-N were carried out at PXI-X06SA using an EIGER 16 M detector. The X-ray wavelength, collection speed, flux and beam size were 1 Å (12.398 keV), 40 deg s^−1^, 2.0–4.0 × 10^11^ photons s^−1^ and 80 × 50 µm, respectively.

The measurements of EP and Thau cryoloop samples treated with paraffin were carried out at PXII-X10SA using an EIGER2 16 M detector. The X-ray wavelength, collection speed, flux and beam size were 1 Å (12.398 keV), 40 deg s^−1^, 2.34 × 10^11^ photons s^−1^ and 75 × 40 µm, respectively.

The 3CL^pro^ data were measured at BM07 (FIP-2), ESRF using a PILATUS 6M detector; the X-raywavelength, collection speed, beam size and flux were 0.9795 Å (12.657 keV), 10 deg s^−1^, 200 × 100 µm and 2.3 × 10^11^ photons s^−1^ for the RT data set and 1.1 × 10^11^ photons s^−1^ for the cryo data set.

### 2.4. Data processing and structure determination

All diffraction data were processed with *XDS* and scaled and merged with *XSCALE* (Kabsch, 2010 ▸). The multi-crystal data sets were selected and merged following a previously described procedure (Basu *et al.*, 2019 ▸). Due to the increased thermal motion, the RT data set has a higher Wilson *B* factor and lower diffraction quality than the cryo data sets. The data-processing statistics of the RT data were used to determine the resolution cutoff, and the same cutoff was applied to the cryo data sets, except for AC39729-IS-RT2 and TL00150-PF-cryo3, where the diffraction resolution is worse than 2 Å.

The structures were solved by molecular replacement using *Phaser* (McCoy *et al.*, 2007 ▸), with PDB entries 7qm6 (Huang *et al.*, 2022 ▸), 1rqw (Q. Ma & G. M. Sheldrick, unpublished work) and 7grr (Huang *et al.*, 2024 ▸) as the search models for EP, Thau and 3CL^pro^, respectively. *BUSTER* version 2.10.4 (Bricogne *et al.*, 2017 ▸) was used for the refinement of all structures, including occupancy refinement. *Coot* (Emsley *et al.*, 2010 ▸) was used for manual structure building. Figures were generated using *PyMOL* version 1.2 (Schrödinger). Structures and the associated structure-factor amplitudes have been deposited in the Protein Data Bank (PDB; Berman *et al.*, 2000 ▸). Data-collection parameters, data-processing and refinement statistics, and PDB accession codes are listed in Supplementary Tables S2–S5.

## 3. Results and discussion

We investigated two sample-delivery methods for RT X-ray data collection from cryo-cooled crystals (Fig. 1 ▸). The first method utilizes a cryo-cooling-compatible IMISX chip (Huang *et al.*, 2016 ▸) to harvest multiple crystals for serial data collection. The second method involves the use of a conventional cryoloop to harvest single crystals, which are subsequently coated with Paratone-N or paraffin oil for typical single-crystal data collection. Both methods were chosen to prevent dehydration during RT measurements (Hope, 1988 ▸; Doukov *et al.*, 2020 ▸).

The unit-cell volume increased by ∼4% from cryo1 to RT2 for EP samples, which is consistent with previous studies (Juers & Matthews, 2001 ▸; Fraser *et al.*, 2011 ▸), with the exception of AC40075 in Paratone-N oil. The latter showed a nearly 12% increase because its cryo structure had a smaller unit cell compared with other cryo structures (Supplementary Fig. S2, Supplementary Tables S2–S4). The cryo1 unit cell was mostly restored for both the IMISX chip and Paratone-N samples in cryo3. However, crystals under paraffin oil might have suffered from dehydration during the RT2 data collection, resulting in a smaller unit-cell volume (Supplementary Fig. S2*c*) and fewer ordered water and DMSO molecules in cryo3 (Supplementary Fig. S3).

### 3.1. The versatile IMISX method captures room-temperature EP structures

The IMISX method was developed for serial data collection at both RT and cryo temperatures and has a format that is compatible with standard sample changers at MX beamlines (Huang *et al.*, 2015 ▸, 2016 ▸). It enables sample preparation at the user laboratory, sample shipping with standard cryo-shippers and robotic mounting at the goniometer, as commonly practiced in cryo-crystallography. While sample preparation with IMISX or similar chips requires additional work compared with harvesting crystals with cryoloops, it has several distinct advantages: (i) the crystals are protected from drying, (ii) serial data collection mitigates radiation damage at RT and is applicable to microcrystals and (iii) both cryo and RT data can be obtained from a single chip, providing a direct comparison of the same condition.

**Figure 2:**
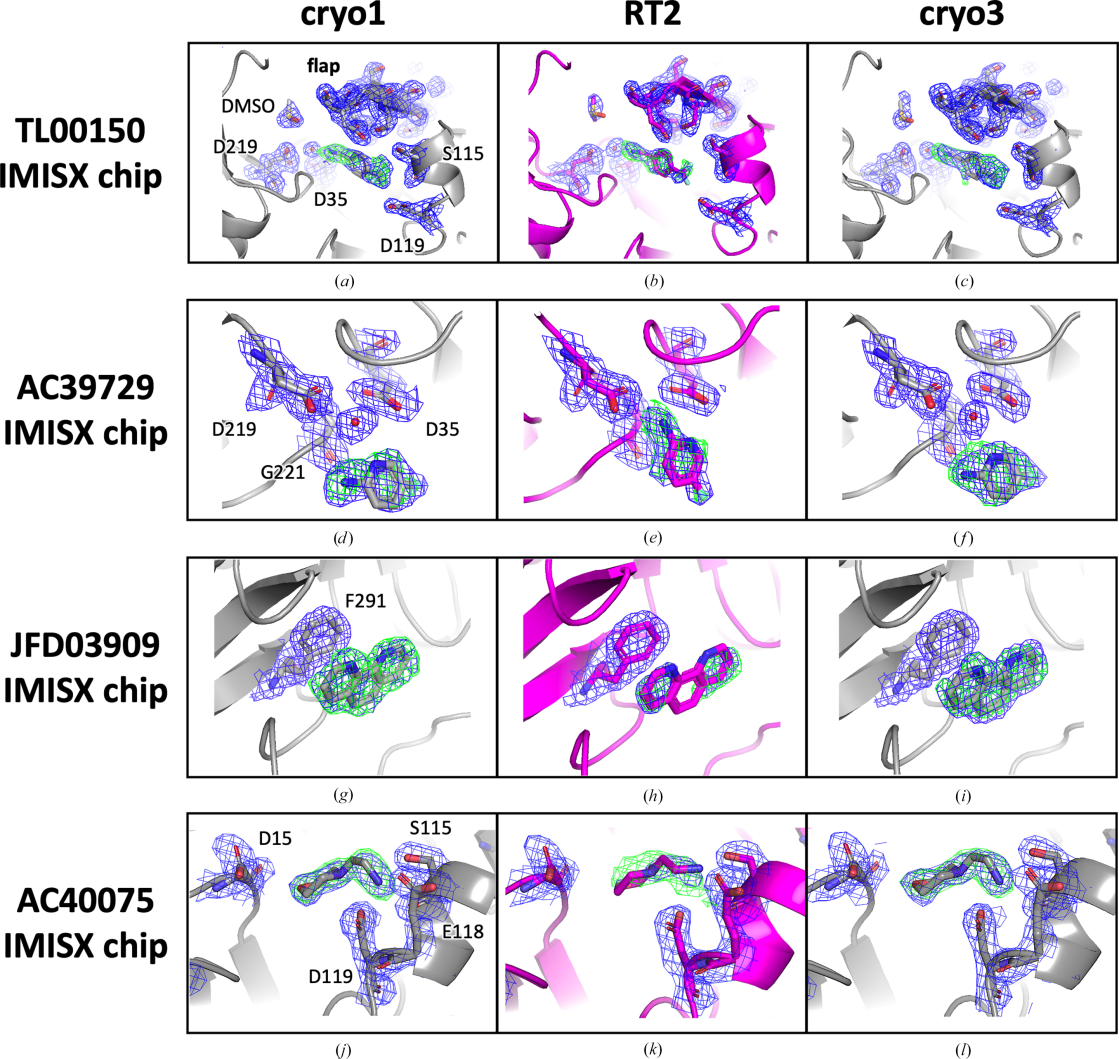
Electron-density maps of structures of EP in complex with TL00150, AC39729, JFD03909 and AC40075 using the IMISX setup under cryo and RT conditions. The EP structure is shown in cartoon representation, and fragments and surrounding residues are shown in stick representation. The cryo and RT structures are colored gray and magenta, respectively. The 2*F*_o_ − *F*_c_ electron-density maps are contoured at the 1.0σ level with blue-colored mesh around the fragment-binding pocket and flap domain (residues Ser76–Ser86) of the EP structures. The *F*_o_ − *F*_c_ electron-density maps generated using the model without the fragment are also shown contoured at the 3.0σ level and colored as a green mesh.

We used medium-sized EP crystals (70 × 30 × 20 µm) grown by batch crystallization and soaked with four fragments (Supplementary Table S1) that had previously been identified by cryo-crystallography fragment-based screening. The cryo1, RT2 and cryo3 data sets were collected from different regions of the same chip. At 100 K (cryo1), TL00150 and AC39729 bound at the S1 pocket near the catalytic residues (Asp35 and Asp219), JFD03909 bound near the entrance to the S2 pocket and AC40075 bound at the S3 pocket (Supplementary Fig. S1). While all of the fragments were bound at 100 K (Figs. 2 ▸*a*, 2 ▸*d*, 2 ▸*g* and 2 ▸*j*), they showed reduced occupancies in RT2 when the crystals were warmed to 296 K (Figs. 2 ▸*b*, 2 ▸*e*, 2 ▸*h* and 2 ▸*k*, and Supplementary Table S6). The process was reversible and the fragments bound back when the crystals were cooled to 100 K (Figs. 2 ▸*c*, 2 ▸*f*, 2 ▸*i* and 2 ▸*l*).

TL00150 exhibits temperature-dependent binding dynamics, as reported previously (Huang *et al.*, 2022 ▸). Briefly, the lower temperature favors binding at the S1 pocket with the flap domain in a closed conformation, and the higher temperature induces an opening motion of the flap domain outwards with respect to the binding pocket and changes the binding site to the S1′ location. We observed this behavior in the current study. At 100 K, TL00150 and DMSO occupied the S1 and S1′ pockets, respectively. Additionally, the flap domain showed a well defined structure, as observed in the 2*F*_o_ – *F*_c_ map (Fig. 2 ▸*a*). At 296 K, the refined occupancy at the S1 pocket was lower (Supplementary Table S6) and the 2*F*_o_ – *F*_c_ density corresponding to the flap domain became less defined, indicating a more flexible behavior of this area compared with that at 100 K (Fig. 2 ▸*b*). After cooling the IMISX chip back to 100 K, the whole process was reversed (Fig. 2 ▸*c*). Using this example, we demonstrated that temperature-dependent ligand binding could be obtained in a single chip from previously cryo-cooled crystals.

AC39729 interacted via a catalytic water at the S1 pocket at 100 K (Fig. 2 ▸*d* and Supplementary Fig. S4). Interestingly, at 296 K it rotated towards the catalytic water by ∼50°, replaced the catalytic water and interacted with the catalytic residues directly (Figs. 2 ▸*e* and 3 ▸*a*). This new binding pose was suppressed when the crystals were cooled back to 100 K (cryo3). Here, the advantage of RT crystallography becomes clear. Without it, the alternative binding mode remains elusive and subsequent modeling and ligand-optimization efforts could be compromised.

In the cryo1 structure, the JFD03909 fragment formed hydrophobic interactions with Phe291 and replaced the solvent molecules (water or PEG) that were initially observed in the 100 K apo structure (PDB entry 7qlw; Fig. 2 ▸*g* and Supplementary Figs. S5*a* and S5*b*). At 296 K, the fragment showed a rather weak density with lower occupancy (Fig. 2 ▸*h*); the JFD03909 fragment then reverted to a higher occupancy in cryo3 (Fig. 2 ▸*i*).

**Figure 3:**
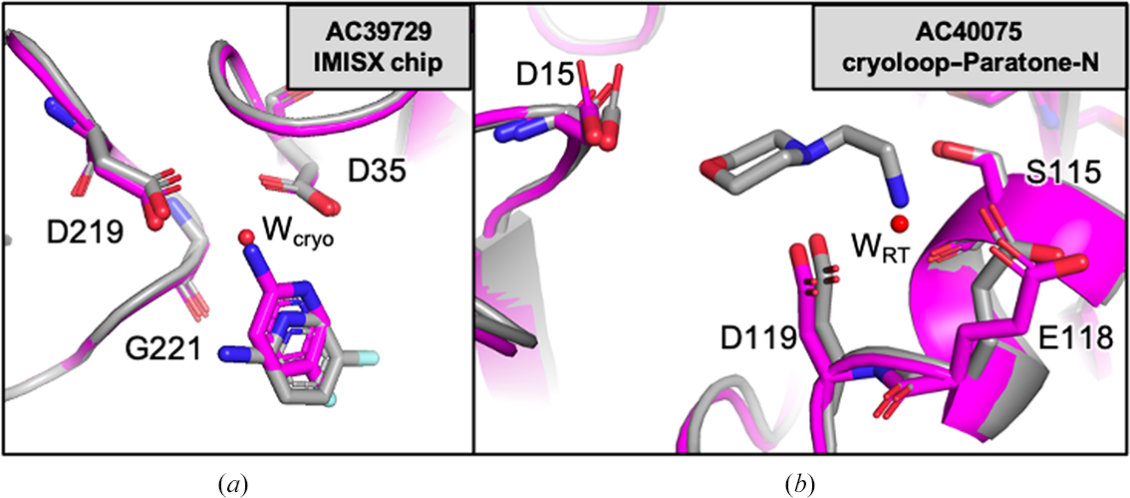
Alignment of cryo1 and RT2 EP structures. (*a*) EP in complex with AC39729 (IMISX chip). W_cryo_ means that the water is only present in the cryo1 structure. (*b*) EP in complex with AC40075 (Paratone-N). W_RT_ means that the water is only present in the RT2 structure. The regions of interest in the cryo1 and RT2 structures are shown in stick representation and colored gray and magenta, respectively.

The AC40075 fragment formed hydrogen bonds to Asp15, Ser115, Glu118 and Asp119 at 100 K and replaced some water molecules that were seen in the apo structure (Fig. 2 ▸*j* and Supplementary Figs. S5*c* and S5*d*). Notably, Ser115, Glu118 and Asp119 exhibited a shift towards the AC40075 fragment (Fig. 3 ▸*b*). Ser115 showed two alternative conformations when no fragment was bound, and Glu118 was oriented towards the solvent in the apo structure (PDB entry 7qlw). At 296 K, the electron density of AC40075 was very weak, but Ser115, Glu118 and Asp119 still pointed towards the fragment, indicating possible lower occupancy (Fig. 2 ▸*k* and Supplementary Table. S6), and the occupancy was restored after cooling to 100 K (Fig. 2 ▸*l* and Supplementary Table. S6).

While the cryo1 and cryo3 structures were nearly identical, exhibiting a root-mean-square deviation (r.m.s.d.) of ∼0.1 Å, the r.m.s.d. between cryo1 and RT2 was slightly higher at ∼0.3 Å (Supplementary Table S7). Variations between the RT2 and cryo structures are primarily observed in the surface-loop regions (Val43–Glu52, Pro254–Phe262, Ile278–Ser289 and Ser296–Asn303) and the flap domain (Tyr79–Gly82) (Supplementary Fig. S6, indicated by red arrows). The most significant loop displacement is found in the Ile278–Ser289 region of EP in complex with AC39729 and AC40075, with a displacement of approximately 1.1 Å.

### 3.2. The high-throughput cryoloop method enables routine RT structure determination

The IMISX method is very suitable for multi-temperature studies of structural dynamics. It is also well suited when only microcrystals can be grown for measurement. However, the average data-collection throughput is considerably lower than in conventional single-crystal cryo-crystallography. For applications where high-throughput structure determination is essential, for example X-ray fragment-based screening at RT, one would need a similar throughput as in cryo-crystallography. Therefore, we set out to perform similar experiments using conventional single crystals mounted with standard cryoloops for three protein targets (EP, Thau and 3CL^pro^). To prevent dehydration during thawing and X-ray data collection, we tried using Paratone-N and paraffin oils to coat the harvested crystals before cryo-cooling. For the RT data collection, the cryostream was blocked and retracted immediately before X-ray data collection (Fig. 1 ▸).

**Figure 4:**
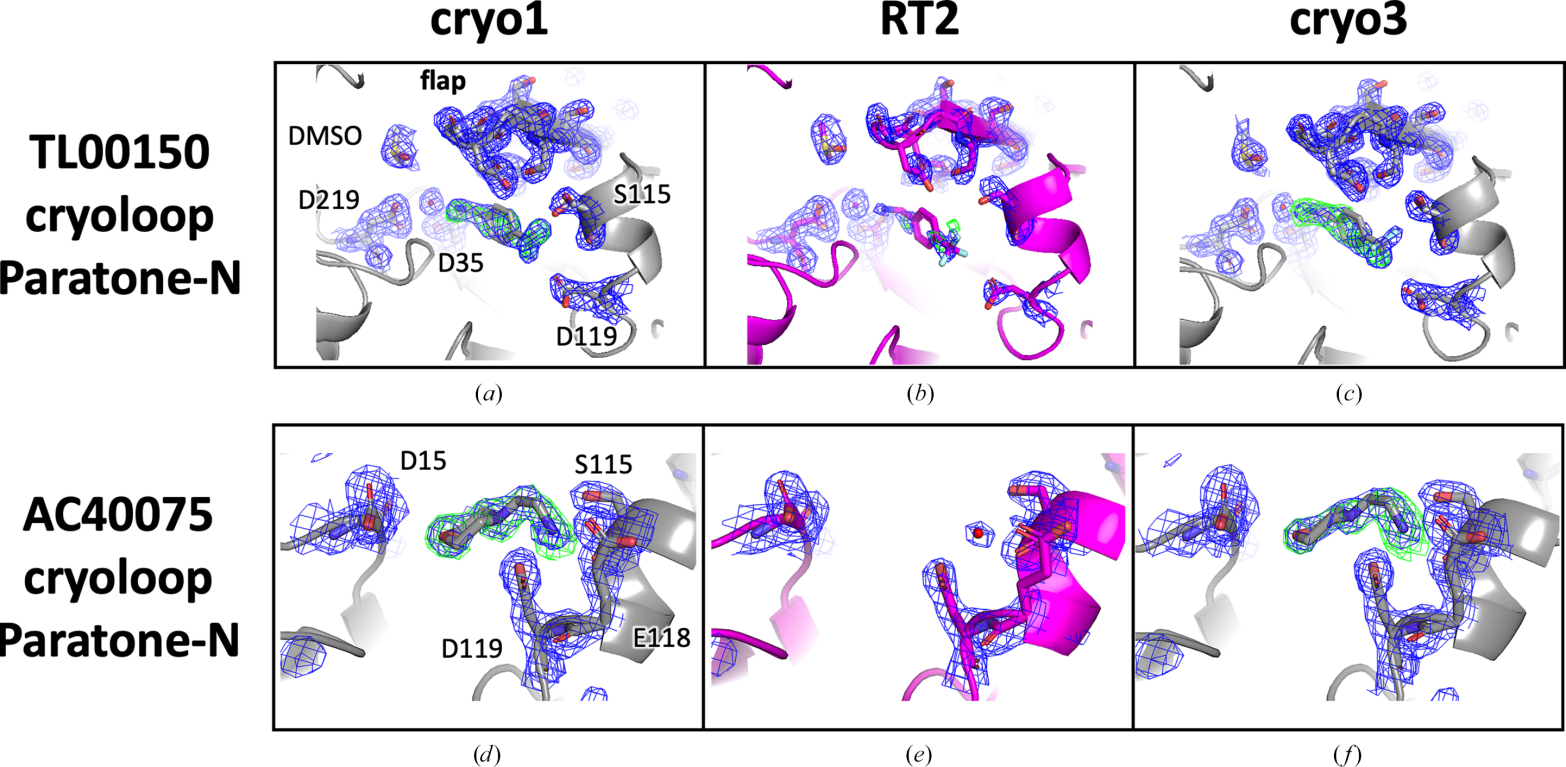
Electron-density maps of structures of EP in complex with TL00150 and AC40075 with the cryoloop–Paratone-N oil setup under cryo and RT conditions. The EP structure is shown in cartoon representation, and fragments and surrounding residues are shown in stick representation. The cryo and RT structures are colored gray and magenta, respectively. The 2*F*_o_ − *F*_c_ electron-density maps contoured at the 1.0σ level with blue-colored mesh around the fragment-binding pocket and flap domain (residues Ser76–Ser86) of the EP structures are shown. The *F*_o_ − *F*_c_ electron-density maps generated using the model without the fragment are also shown contoured at the 2.5σ level and colored green.

Two fragments, TL00150 and AC40075, were used with Paratone-N oil-coated EP crystals. We obtained similar results as those observed with the IMISX method at 100 K (Fig. 4 ▸). At 296 K, the fragment occupancy at the S1 pocket was reduced for TL00150. At 296 K, AC40075 was released from the S3 pocket and replaced by a water molecule, and Glu118 became disordered (Figs. 3 ▸*b* and 4 ▸*e*).

The cryo1 and cryo3 structures were almost identical, with an r.m.s.d. value of 0.1 Å, and variations between the RT2 and cryo structures were primarily observed in the surface-loop regions, as observed with the IMISX chip sample (Supplementary Table S6 and Supplementary Fig. S6, indicated by red arrows). Particularly noteworthy is the difference in the loop region Ile278–Ser289, with a displacement of approximately 2.0 Å of the cryoloop sample of EP in complex with AC40075.

With paraffin oil, all four fragments were bound in the cryo1 structures (Supplementary Figs. S7*a*, S7*d*, S7*g* and S7*j*). However, JFD03909 and AC40075 disappeared in the RT2 structures and TL00150 and AC39729 were replaced by ‘unexplained’ electron densities at the active site (Supplementary Figs. S7*b*, S7*e*, S7*h* and S7*k*). On cooling to cryo3, none of the fragments bound back (Supplementary Figs. S7*c*, S7*f*, S7*i* and S7*l*). Taking into account the reduced unit-cell volume compared with Paratone-N oil samples in cryo3 (Supplementary Fig. S2), paraffin oil may have interfered with fragment binding during thawing/cooling and is not a suitable choice for this experiment.

Cryo2RT was also applied to Thau and 3CL^pro^ cryoloop crystals, yielding high-quality data sets after the cooling/thawing process (Supplementary Table S5). Using this approach, we showed that the cryo-crystallography throughput is achievable, and more importantly, that the well established cryo-crystallography practices can be applied to obtain RT (or higher temperature in general) structures.

### 3.3. Opportunities and limitations

Cryo-condition optimization is a standard practice in cryo-crystallography, but less attention has been paid to the thawing process, which could introduce crystalline ice into the crystal solvent channels and mother liquor surrounding the crystals and damage the crystals (Kriminski *et al.*, 2002 ▸). The impact of cooling/thawing cycles on crystalline order, lattice strain, domain volume and orientation, and diffraction resolution has only been studied carefully on a few proteins (Kriminski *et al.*, 2002 ▸; Juers & Matthews, 2004*a* ▸,*b* ▸; Juers *et al.*, 2007 ▸; Moreau *et al.*, 2019 ▸). Therefore, one obvious limitation of the proposed Cryo2RT method is that not all protein crystals can survive the freezing/thawing cycle. Fortunately, crystals for RT measurements from fragment-based screening projects are often robust and well diffracting, as are the crystals used in this study. As more protein systems are studied with Cryo2RT, it will be beneficial to further optimize the cooling/thawing process to make the methods more generally applicable.

In this study, we systematically observed a slightly lower resolution for crystals collected at RT compared with cryo temperature, even though the same dose of a few hundred kilograys was used (Supplementary Figs. S8–S11). A plausible explanation is the increased thermal motion at RT (Thorne, 2023 ▸). For EP, the cryo1 structures have a *B* factor of ∼20 Å^2^, while the RT structures have a *B* factor of 30–40 Å^2^ for the same 2 Å resolution cutoff. This agrees with the temperature-dependent behavior of *B* factors reported previously (de Sá Ribeiro & Lima, 2023 ▸).

In this demonstration work, we had a glimpse of the impact of cryo-cooling on fragment binding. The methods can readily be extended to multiple temperatures by varying the cryostream temperature, offering easy access to temperature-dependent studies of protein conformation, structural heterogeneity and ligand-binding processes (Keedy *et al.*, 2015 ▸, 2018 ▸; Horrell *et al.*, 2018 ▸). On the other hand, if the main purpose is to obtain RT structures, one could forgo the cryogenic data collection (cryo1), let the crystal thaw at the goniometer without a cryostream and collect RT data directly. This further improves the throughput. In addition, the proposed workflow allows the samples to be retrieved back in the ‘cryo state’ and stored in the shipping Dewar after RT data collection, albeit with a slow cryo-cooling process using a cryo-gripper of the sample changer. In this way, samples may be reused for further investigations with cryo-crystallography provided that they survive this process.

Two sample-delivery methods have been tried in this study, and both could be further improved. Essentially, any enclosed chip with a form factor compatible with sample changers and cryostreams at synchrotron beamlines could be used. Modifications to the chip structure, as well as the crystal-loading step, could minimize the crystal settling time upon thawing. The delicate nature of protein crystals and the need for dexterity in the oil-coating process, as well as in screening, or the use of dehydration-proof oil can make this demanding and time-consuming. Additionally, attention needs to be paid to minimizing possible dehydration during cryoloop data collection, which could affect the crystal resolution and produce conformational changes (Atakisi *et al.*, 2018 ▸). One solution is to use a humidifier jet to keep the crystal hydrated during X-ray data collection. Such devices are commercially available and can also be custom-built for specific sample environments at synchrotron beamlines (Kiefersauer *et al.*, 2000 ▸; Sjögren *et al.*, 2002 ▸; Sanchez-Weatherby *et al.*, 2009 ▸; Baba *et al.*, 2019 ▸). The crystal drop may shrink or swell depending on the crystallization solution and the controlled humidity, but fast X-ray data collection can deliver a complete diffraction data set in seconds before significant disturbance occurs, thanks to the use of the newer generation of X-ray detectors operated at a kilohertz frame rate at fourth-generation synchrotron sources (Chapman, 2018 ▸, 2023 ▸).

In recent ligand-screening studies at RT (Skaist Mehlman *et al.*, 2023 ▸), decreased hits at RT are a recurring observation. We observed the same in the current study. The thermodynamics of ligand binding could manifest in various ways in crystal structures determined at different temperatures. The method described here facilitates RT ligand screening and enables systematic studies under both cryo and RT conditions. Therefore, contemporary RT methods will complement the conventional cryo methods. Cryo structures will allow us to identify weaker binders, and RT structures may provide more physiologically relevant binding poses. Such studies on a wide range of protein targets will also provide insights into the discrepancies that are often observed between biophysical binding assays at RT and hits in cryo-crystallography.

Another interesting observation is that the cryo structures obtained from IMISX chips and cryoloops exhibit nearly identical features. However, there are slight discrepancies between the RT structures obtained using the IMISX and cryoloop methods, particularly in the fragment occupancy and the local arrangement of DMSO and water molecules. This suggests that cryo-cooling tends to favor low-enthalpy states and may selectively stabilize one conformation from the intrinsic ‘dynamic structures’ that are present at RT. Since RT structures are influenced by various experimental conditions such as the solvent around the crystal, the crystal size and the presence of protective oils, it is necessary to conduct multiple RT structure determinations and employ ensemble-refinement techniques (Ploscariu *et al.*, 2021 ▸; Du *et al.*, 2023 ▸) for each target in addition to the standard refinement protocols (Sielecki *et al.*, 1979 ▸; Tronrud, 2004 ▸). Indeed, the presence of ensemble structures and structural heterogeneity is a characteristic feature of RT structures (Fraser *et al.*, 2011 ▸; Mokhtari *et al.*, 2021 ▸; Yabukarski *et al.*, 2022 ▸). We anticipate that our Cryo2RT method will allow user-friendly access to the determination of multiple-temperature structures, thereby enhancing our understanding of structural heterogeneities and their functional significance in protein function and catalysis.

Finally, but importantly, high-resolution RT structures are highly desirable in the era of artificial intelligence and machine-learning-based structure prediction and modeling. Until now, such models have predominantly relied on cryo structures (Jumper *et al.*, 2021 ▸). However, the availability of accurate RT ligand-bound structures holds great promise for structure-based rational design and drug-discovery efforts. Moving beyond cryo ‘static’ structures, the determination of structural ensembles at multiple temperatures offers experimental insights into the energy landscape. This in turn could pave the way for improved modeling and prediction of protein and binding dynamics (Hekstra, 2023 ▸; Fraser & Murcko, 2024 ▸).

## Supplementary Material

PDB reference: EP-AC39729-PF-cryo3, 7h56

PDB reference: EP-AC39729-PF-cryo1, 7h57

PDB reference: EP-AC39729-PF-RT2, 7h58

PDB reference: EP-AC39729-IS-cryo3, 7h59

PDB reference: EP-AC39729-IS-cryo1, 7h5a

PDB reference: EP-AC39729-IS-RT2, 7h5b

PDB reference: EP-AC40075-PN-cryo3, 7h5c

PDB reference: EP-AC40075-PN-cryo1, 7h5d

PDB reference: EP-AC40075-PN-RT2, 7h5e

PDB reference: EP-AC40075-PF-cryo3, 7h5f

PDB reference: EP-AC40075-PF-cryo1, 7h5g

PDB reference: EP-AC40075-PF-RT2, 7h5h

PDB reference: EP-AC40075-IS-cryo3, 7h5i

PDB reference: EP-AC40075-IS-cryo1, 7h5j

PDB reference: EP-AC40075-IS-RT2, 7h5k

PDB reference: EP-TL00150-PN-cryo3, 7h5l

PDB reference: EP-TL00150-PN-cryo1, 7h5m

PDB reference: EP-TL00150-PN-RT2, 7h5n

PDB reference: EP-TL00150-PF-cryo3, 7h5o

PDB reference: EP-TL00150-PF-cryo1, 7h5p

PDB reference: EP-TL00150-PF-RT2, 7h5q

PDB reference: EP-TL00150-IS-cryo3, 7h5r

PDB reference: EP-TL00150-IS-cryo1, 7h5s

PDB reference: EP-TL00150-IS-RT2, 7h5t

PDB reference: EP-JFD03909-PF-cryo3, 7h5u

PDB reference: EP-JFD03909-PF-cryo1, 7h5v

PDB reference: EP-JFD03909-PF-RT2, 7h5w

PDB reference: EP-JFD03909-IS-cryo3, 7h5x

PDB reference: EP-JFD03909-IS-cryo1, 7h5y

PDB reference: EP-JFD03909-IS-RT2, 7h5z

PDB reference: Thau-PF-cryo, 9fx4

PDB reference: Thau-PF-RT, 9fx5

PDB reference: 3CL-PN-cryo, 9fx6

PDB reference: 3CL-PN-RT, 9fx7

Supplementary Figures and Tables. DOI: 10.1107/S2059798324006697/rr5245sup1.pdf
