# Supplementary material for: Cryo2RT: a high-throughput method for room-temperature macromolecular crystallography from cryo-cooled crystals
Source: Acta Crystallogr D Struct Biol. 2024 Jul 25;80(Pt 8):620–8. doi: 10.1107/S2059798324006697 (PMC11301757; doi:10.1107/S2059798324006697)
Supplement: Supplementary file 1 [file d-80-00620-sup1.pdf]

STRUCTURAL
BIOLOGY

Volume 80 (2024)

Supporting information for article:

Cryo2RT: a high-throughput method for room-temperature macromolecular crystallography from frozen crystals

Chia-Ying Huang, Sylvain Aumonier, Vincent Olieric and Meitian Wang

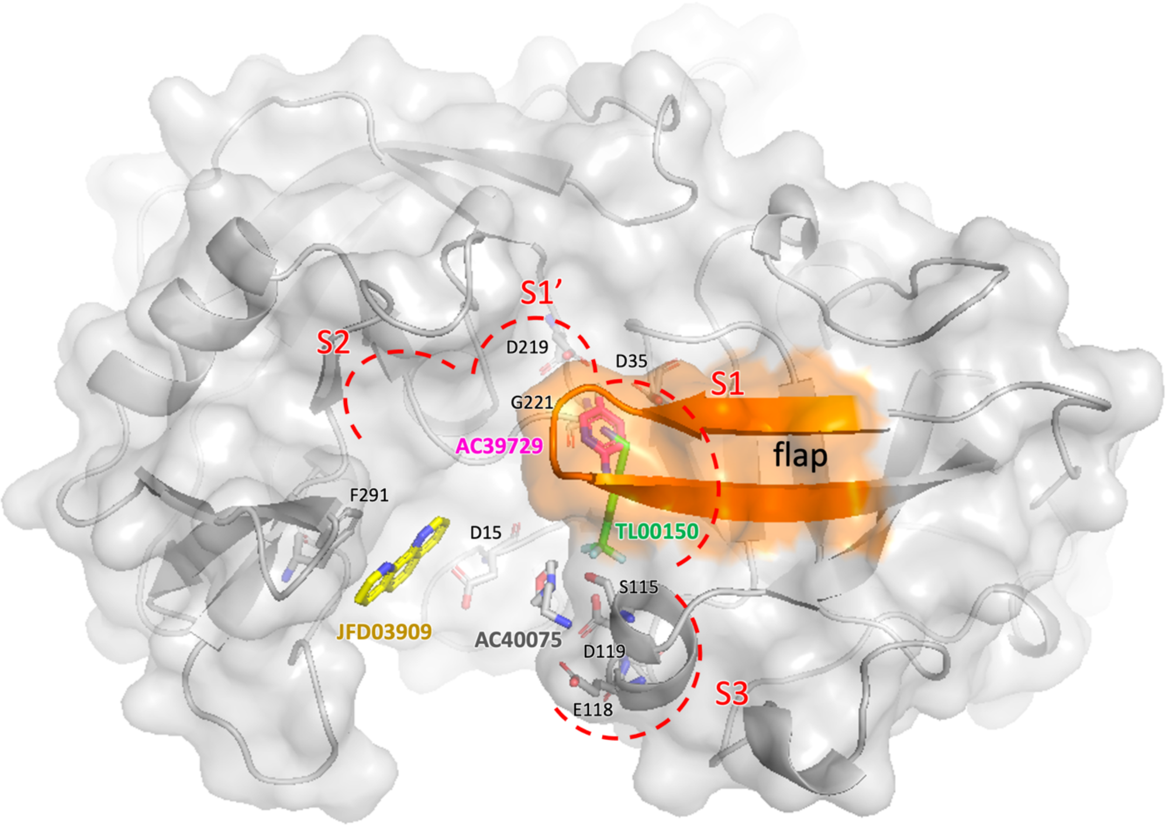

Figure S1. EP binding pockets with fragments and interacting residues. The EP structure is shown in both cartoon and surface representation, colored in gray and transparent gray, respectively. The TL00150, AC39729, JFD03909, and AC40075 fragments are shown in stick representation and colored in green, magenta, yellow, and gray, respectively. The EP residues close to the fragments are represented as sticks and colored in gray. Red dashed semicircles indicate the S1, S1’, S2, and S3 binding pockets of the EP. The flap domain is highlighted in orange.

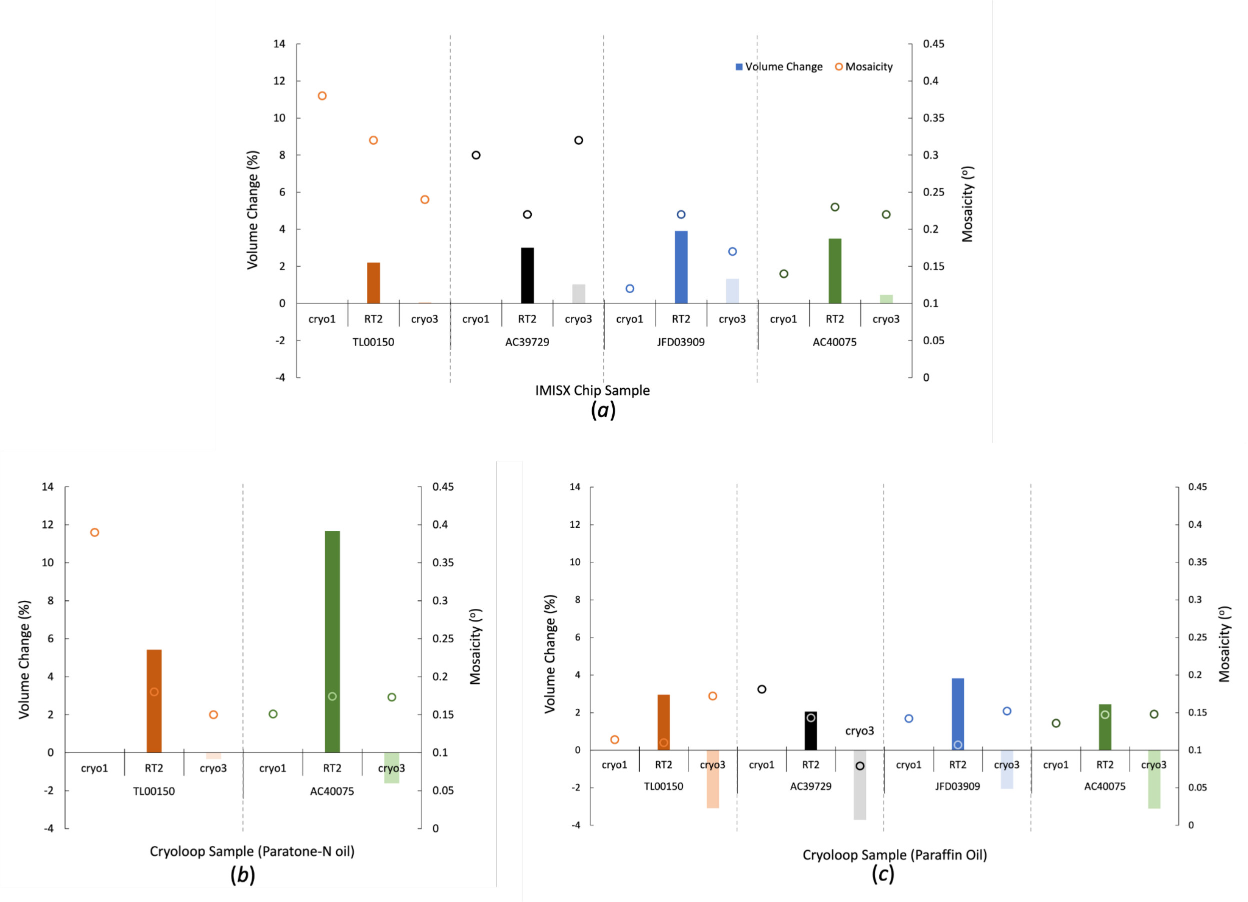

Figure S2. **Changes in unit cell volume and mosaicity of EP**. Volume and mosaicity changes are shown by the bar and open circle, respectively. The percentage changes in unit cell volume were normalized to cryo1.

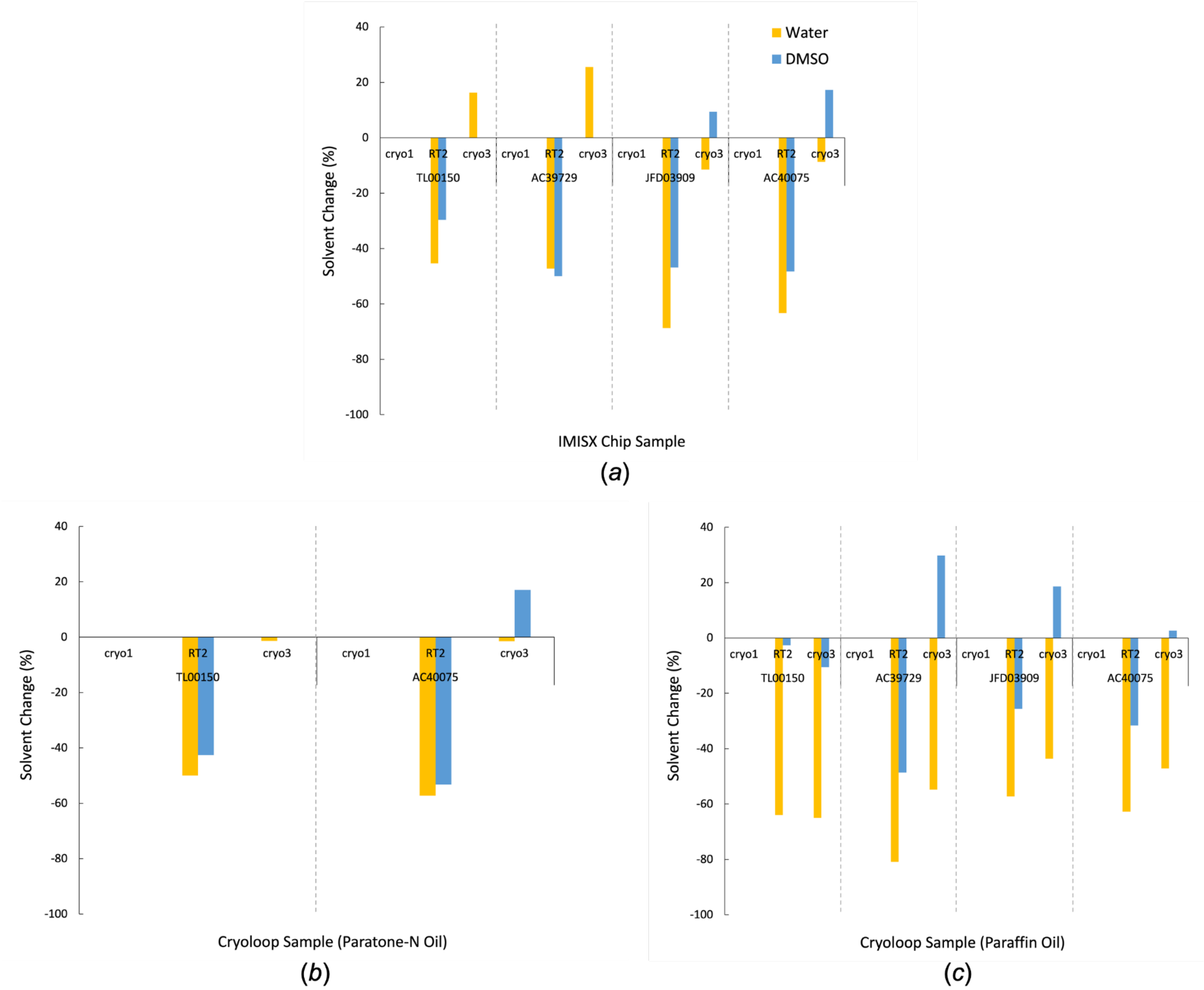

Figure S3. **Changes in observed water and DMSO molecules of EP structures**. The percentage changes in solvent were normalized to cryo1.

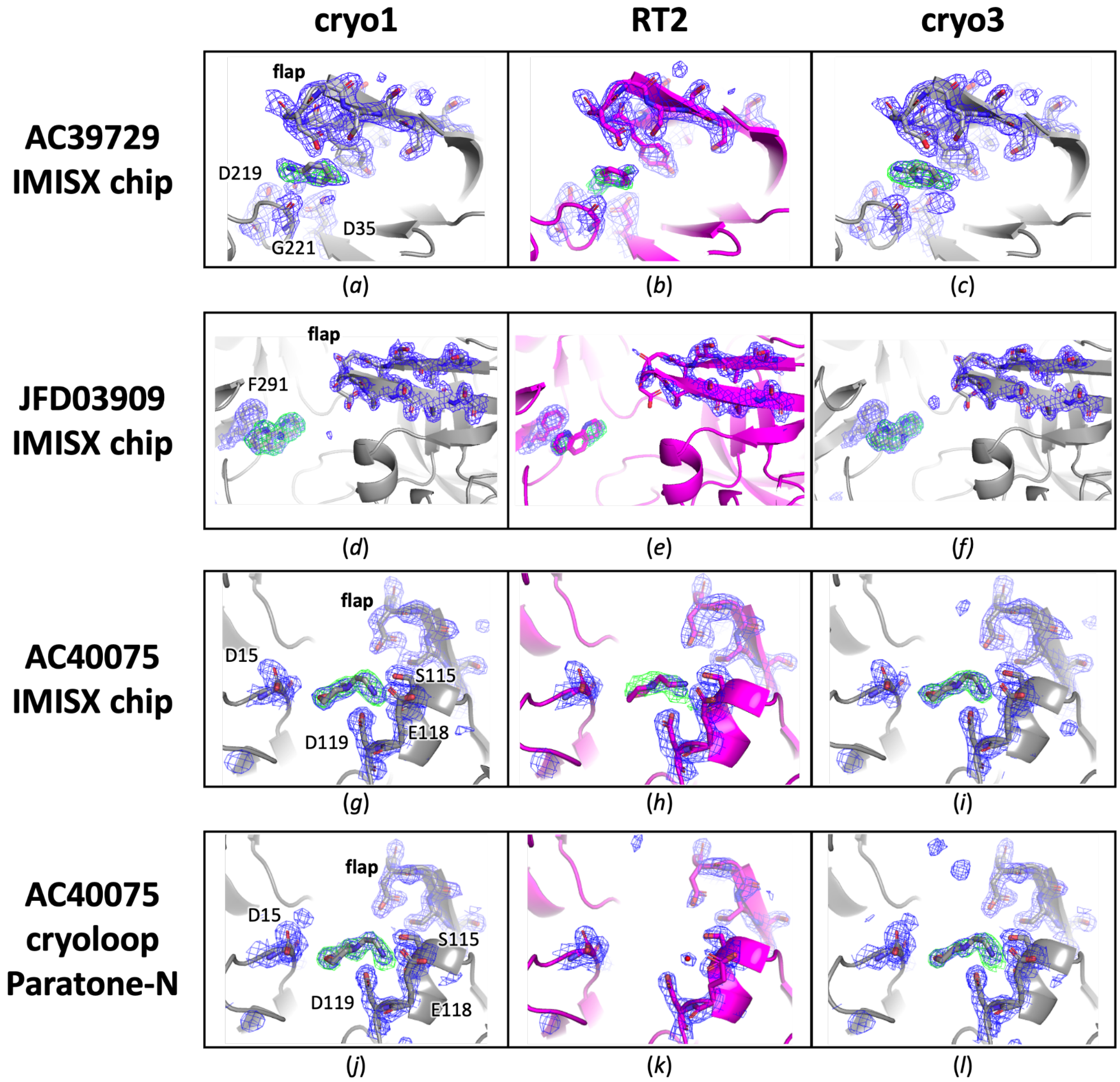

Figure S4. Electron density maps around the flap domain of the EP structures in complex with AC39729, JFD03909, and AC40075, respectively, at cryo and RT conditions. (a)-(i) The samples are set up with IMISX chips. (j)-(l) The samples are set up with cryoloop. The structure is shown in cartoon representation, and fragments and surrounding residues are shown in stick representation. The cryo and RT structures are colored in gray and magenta, respectively. The $2Fo - Fc$ electron density maps contoured at a 1.0 σ level with blue colored mesh around the fragment binding pocket and the flap domain (residues S76 to S86) of EP structures are shown. The $Fo - Fc$ electron density maps generated by the model without the fragment are also shown, contoured at a 3.0 σ level for (a)-(i) and 2.5 σ level for (j)-(l) and colored green on the fragment.

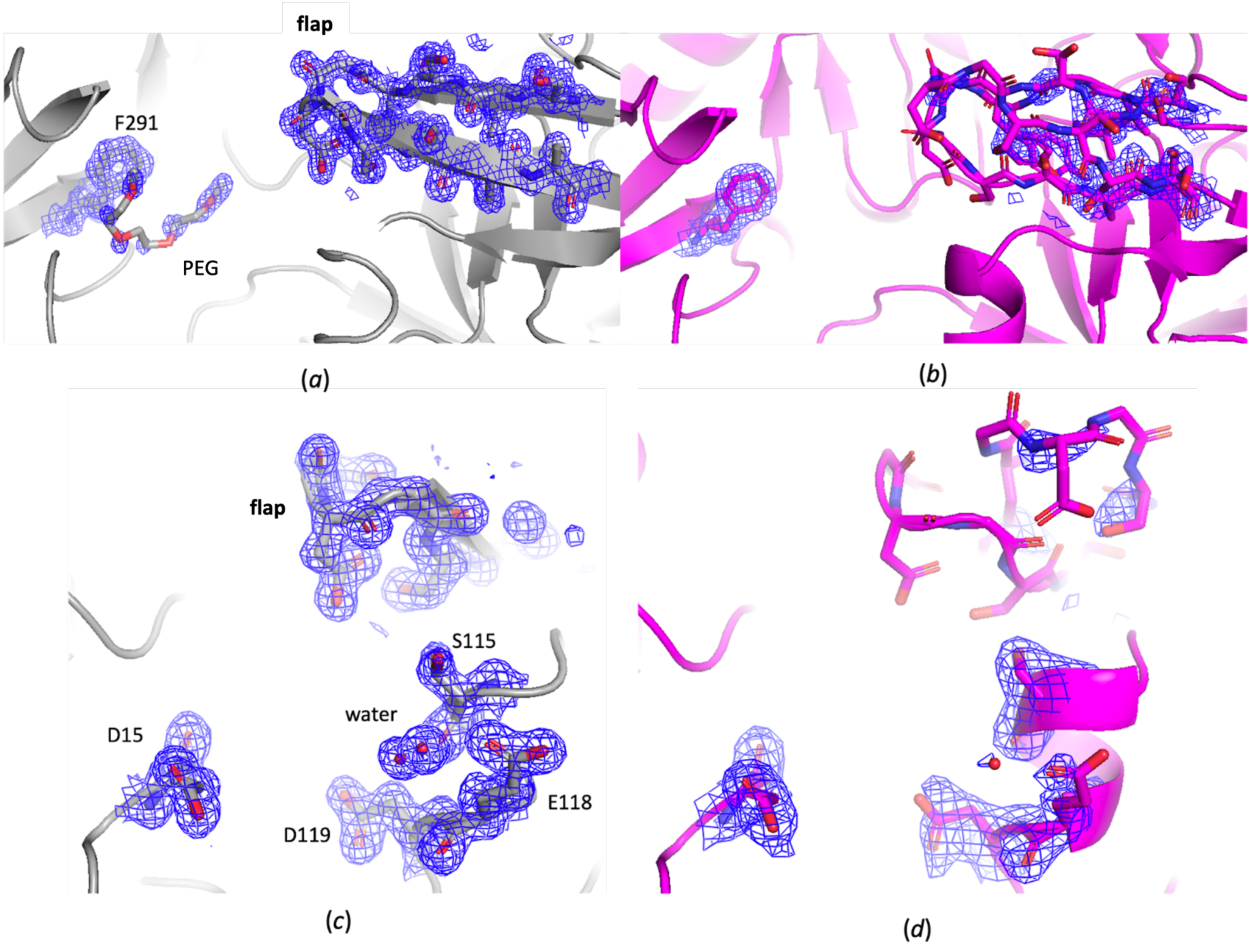

Figure S5. Electron density maps of the EP apo structures with 10% DMSO at cryo (PDB code 7QLW) and RT (PDB code 7QM0) conditions. The structure is shown in cartoon and stick representation, and fragment-related surrounding residues are shown in stick representation. The cryo and RT structures are colored in gray and magenta, respectively. The 2*Fo* – *Fc* electron density maps contoured at 1.0 σ level with blue colored mesh around the fragment binding pocket and the flap domain (residues S76 to S86) of EP structures are shown. (a) and (b) show the binding pocket of the JFD03909 at cryo and RT, respectively. (c) and (d) show the binding pocket of the AC40075 at cryo and RT, respectively.

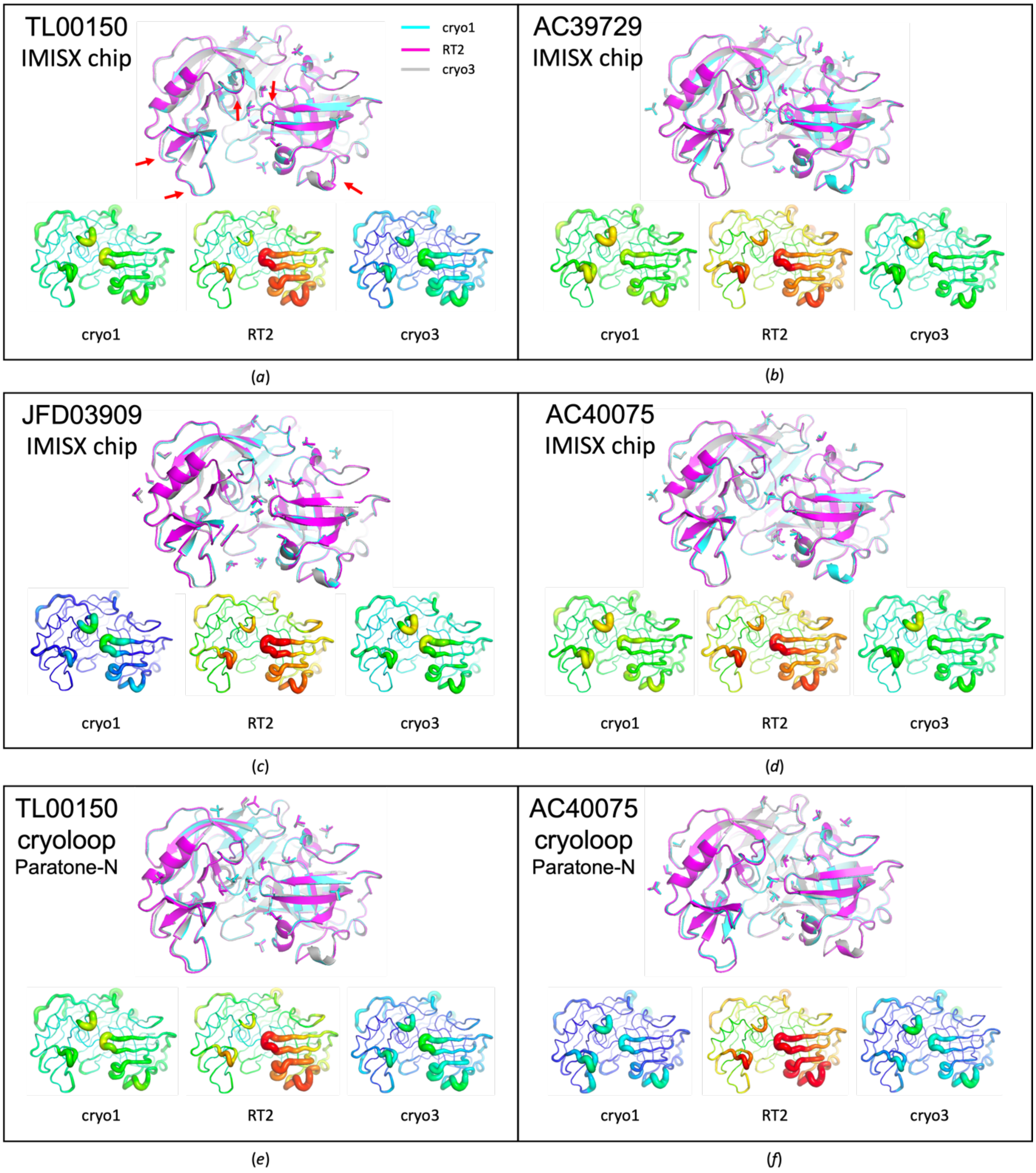

Figure S6. Structure comparison and B-factor distribution of the EP structures in complex with TL00150, AC39729, JFD03909, and AC40075, respectively, at cryo and RT conditions. (*a*-*d*) IMISX chip. (*e* and *f*) cryoloop. The alignment was made by aligning the backbones of EP, and the color codes are depicted in (a), which applied for (*b*-*f*) as well. The red arrows shown in (*a*) and also applied (but not pictured) to (*b*-*f*) indicated the major differences between structures. The B-factors across the structure are also specified for each EP-fragment complex. The B-factor color code ranges from blue to red, indicating low to high (minimum = 10 and maximum = 60) B-factors, respectively.

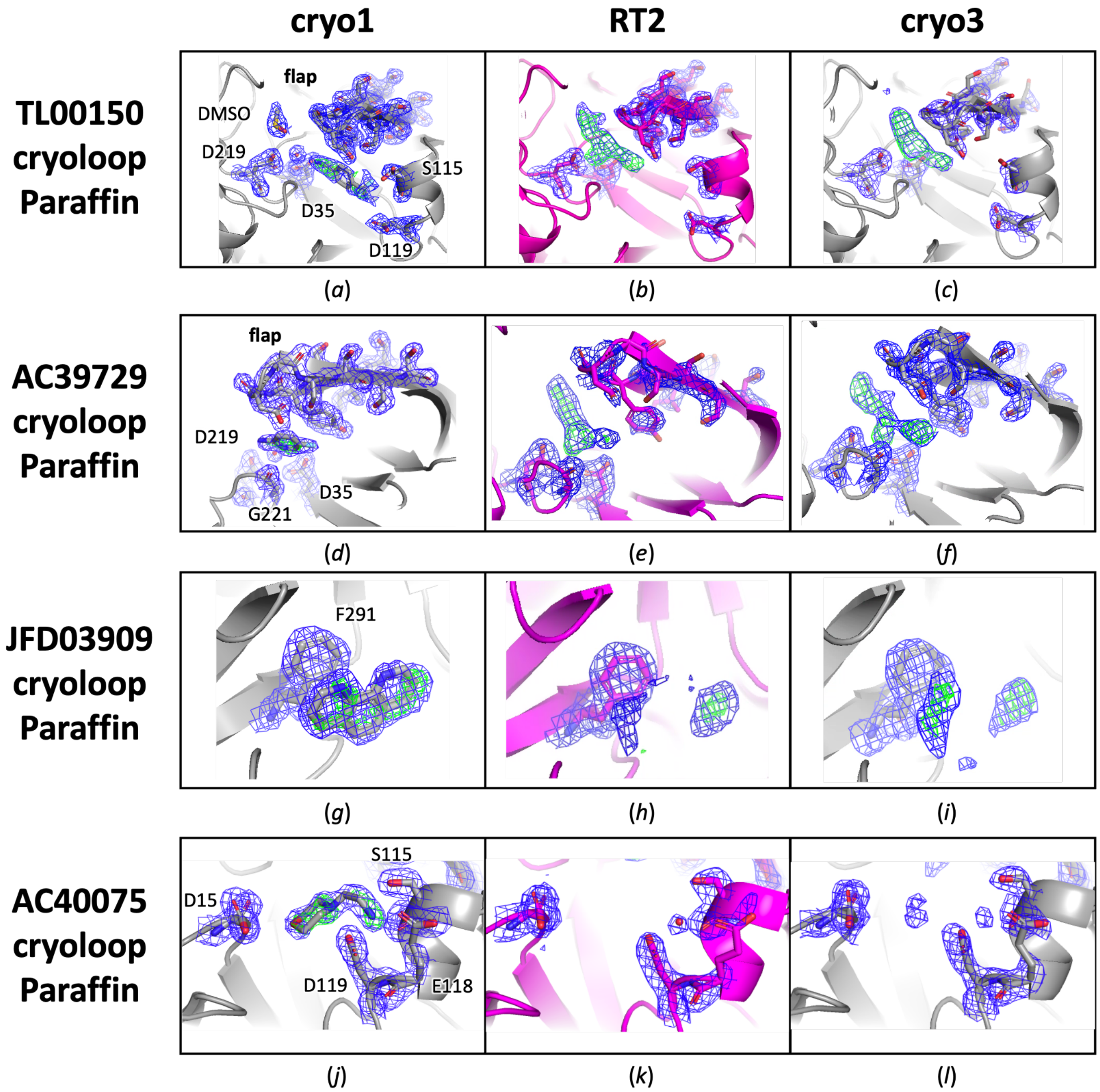

Figure S7. Electron density maps of the EP structures in complex with TL00150, AC39729, JFD03909, and AC40075, respectively, with cryoloop-paraffin oil setup at cryo and RT conditions. The structure is shown in cartoon representation, and fragments and surrounding residues are shown in stick representation. The cryo and RT structures are colored in gray and magenta, respectively. The 2*Fo* – *Fc* electron density maps contoured at 1.0 σ level with blue colored mesh around the fragment binding pocket and the flap domain (residues S76 to S86) of EP structures are shown. The *Fo* – *Fc* electron density maps generated by the model without the fragment are also shown, contoured at a 2.5 σ level, and colored with a green mesh on the fragment.

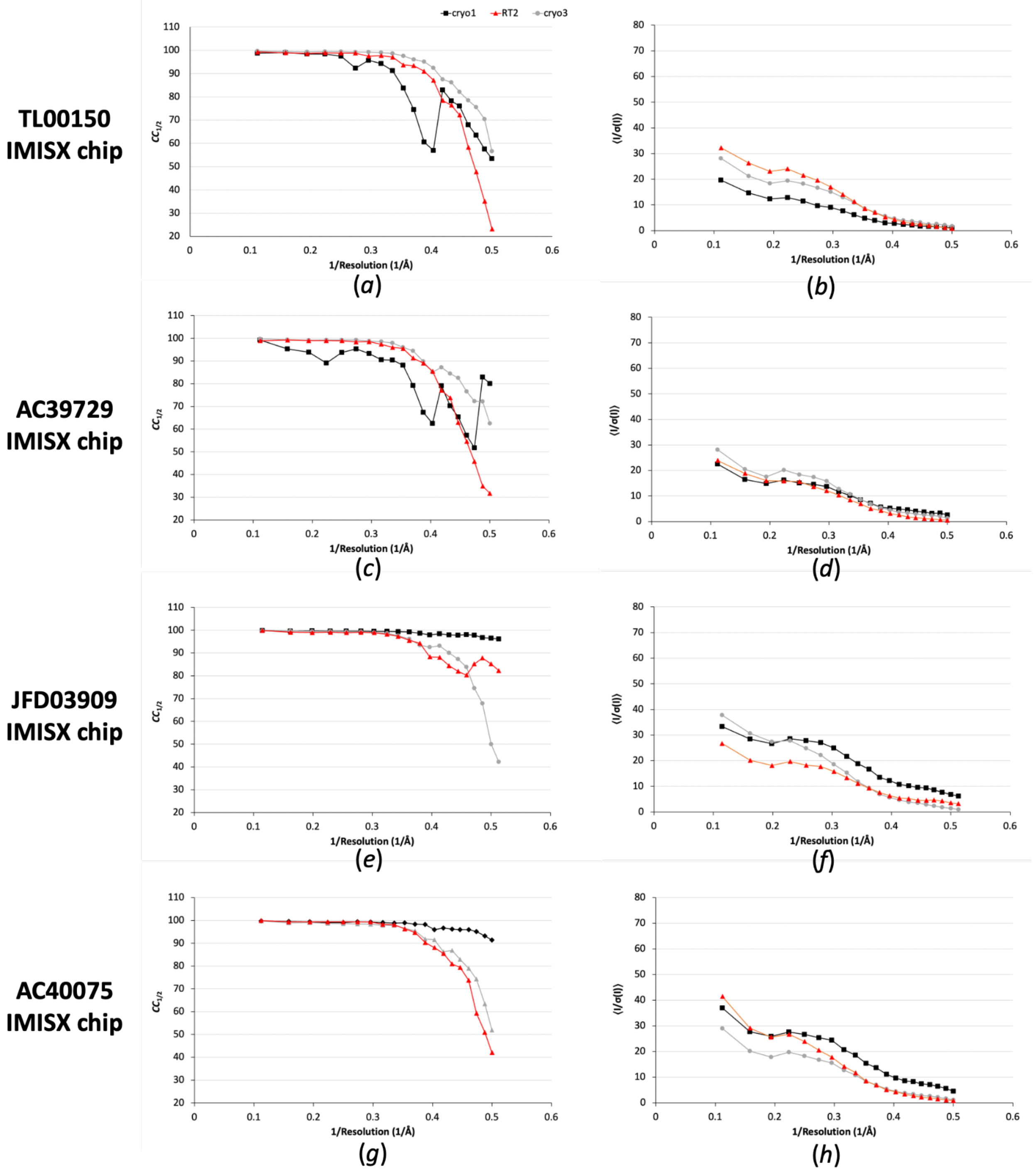

Figure S8. Comparison of data processing statistics of EP structures from IMISX chip. Each row shows the data from the same sample as indicated on the left. The left panel shows $CC_{1/2}$ *v.s.* 1/Resolution and the right panel shows ⟨I/σ(I)⟩ *v.s.* 1/Resolution.

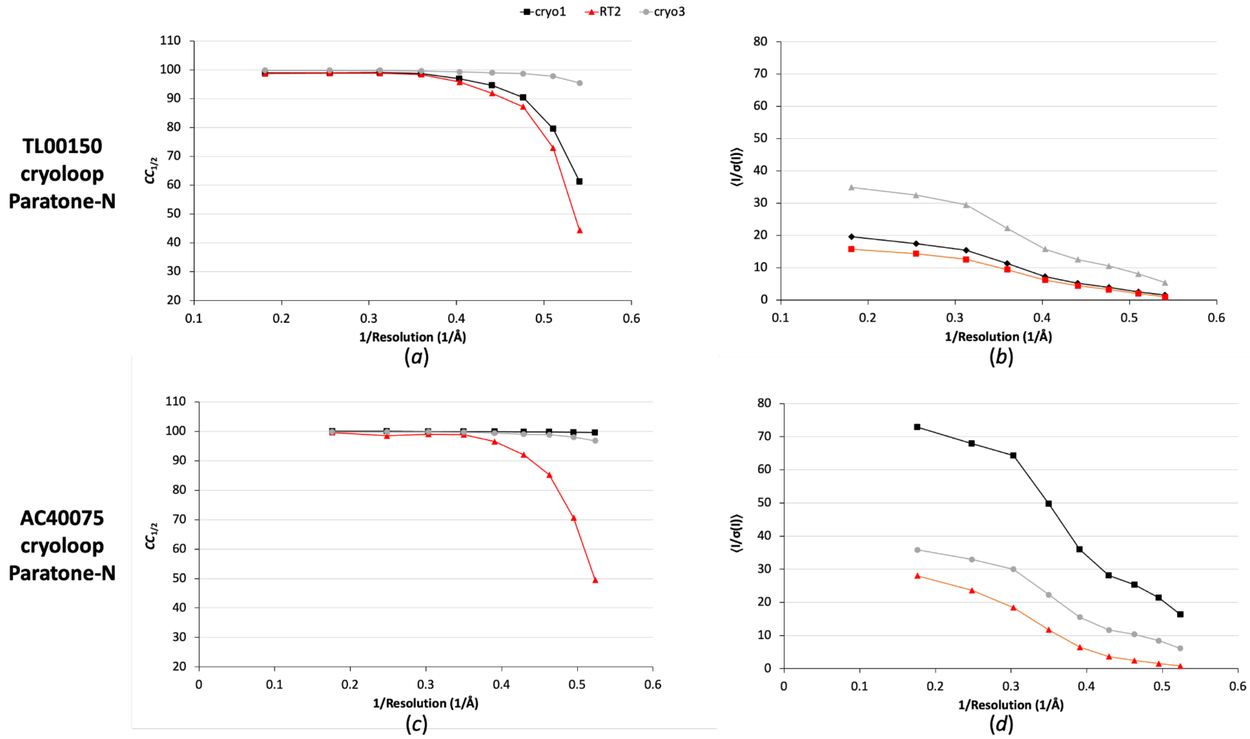

Figure S9. Comparison of data processing statistics of EP Paratone-N structures. Each row shows the data from the same sample as indicated on the left. The left panel shows $CC_{1/2}$ *v.s.* 1/Resolution and the right panel shows ⟨I/σ(I)⟩ *v.s.* 1/Resolution.

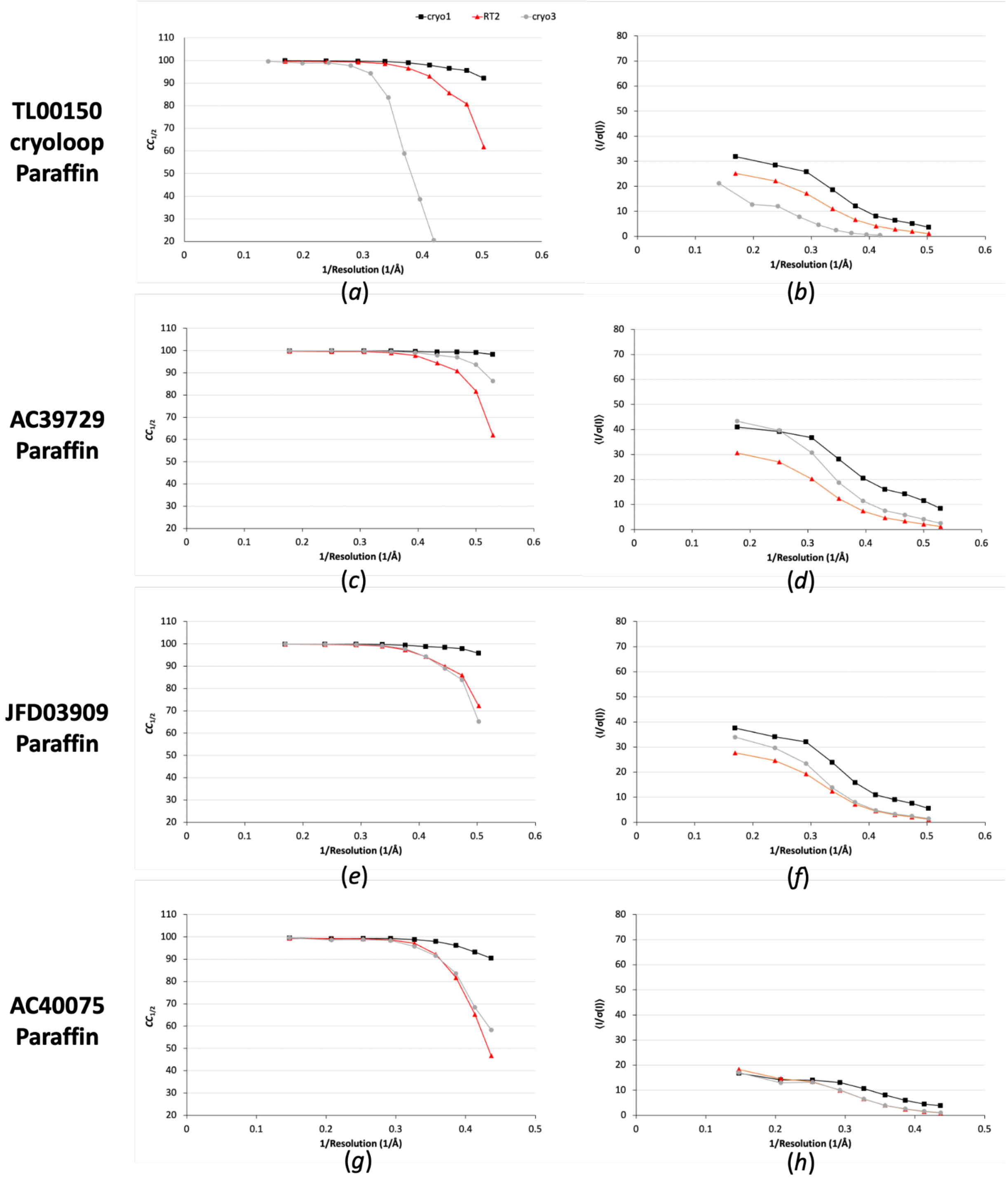

Figure S10. Comparison of data processing statistics of EP Paraffin structures. Each row shows the data from the same sample as indicated on the left. The left panel shows $CC_{1/2}$ *v.s.* 1/Resolution and the right panel shows ⟨I/σ(I)⟩ *v.s.* 1/Resolution.

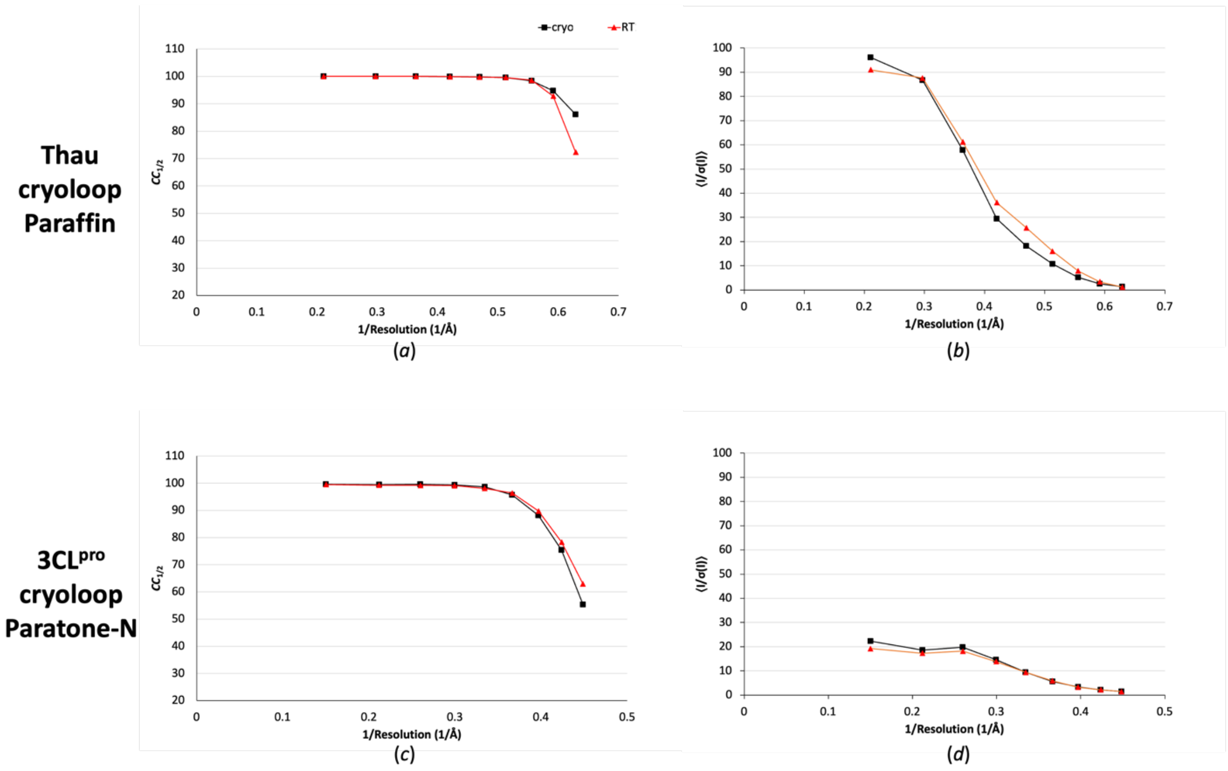

Figure S11. Comparison of data processing statistics of Thau-Paraffin and 3CL^{pro} Paratone-N structures. Each row shows the data from the same sample as indicated on the left. The left panel shows $CC_{1/2}$ *v.s.* 1/Resolution and the right panel shows ⟨I/σ(I)⟩ *v.s.* 1/Resolution.

Table S1. Fragment specification

Fragment code	Full name	SMILES	Chemical structure*
TL00150	4(trifluoromethyl)benzylamine	NCc1ccc(C(F)(F)F)cc1	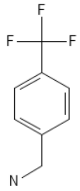
AC39729	2-Amino-5-fluoropyridine	Nc(cc1)ncc1F	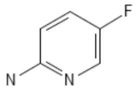
JFD03909	1,10-Phenanthroline monohydrate	c1cc2ccc(cccn3)c3c2nc1	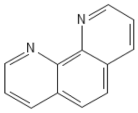
AC40075	4-Morpholineethanamine	NCCN1CCOCC1	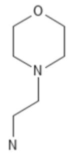

*Chemical structure was generated with PubChem (https://pubchem.ncbi.nlm.nih.gov/#draw=true)

Table S2. Statistic of the EP-fragment IMISX (IS) structures

	TL00150-IS-cryo1	TL00150-IS-RT2	TL00150-IS-cryo3	AC39729-IS-cryo1	AC39729-IS-RT2	AC39729-IS-cryo3	JFD03909-IS-cryo1	JFD03909-IS-RT2	JFD03909-IS-cryo3	AC40075-IS-cryo1	AC40075-IS-RT2	AC40075-IS-Cryo3
**PDB code**	7H5S	7H5T	7H5R	7H5A	7H5B	7H59	7H5Y	7H5Z	7H5X	7H5J	7H5K	7H5I
**Data collection****												
**Temperature (K)**	100	296	100	100	296	100	100	296	100	100	296	100
**Sample delivery**	IMISX Chip	IMISX Chip	IMISX Chip	IMISX Chip	IMISX Chip	IMISX Chip	IMISX Chip	IMISX Chip	IMISX Chip	IMISX Chip	IMISX Chip	IMISX Chip
**No. of crystals**	7	7	8	8	5	7	9	4	11	6	5	6
**Total rotation range (°)**	610	630	600	490	500	480	430	440	450	590	650	600
**Space group**	$P2_1$	$P2_1$	$P2_1$	$P2_1$	$P2_1$	$P2_1$	$P2_1$	$P2_1$	$P2_1$	$P2_1$	$P2_1$	$P2_1$
**Unit-cell parameters**												
a, b, c **(Å)**	45.56, 73.98, 53.17	45.96, 74.18, 53.71	45.46, 73.96, 54.34	45.35, 74.03, 53.06	45.98, 74.22, 53.76	45.51, 74.02, 53.29	45.26, 73.72, 52.89	46.055, 74.13, 53.71	45.40, 74.00, 53.22	45.40, 73.72, 53.09	45.97, 74.12, 53.70	45.42, 73.82, 53.24
**β (°)**	109.82	109.93	110.14	109.88	109.97	110.12	109.78	109.87	109.98	109.9	109.76	109.99
**Mosaicity (°)**	0.38	0.32	0.24	0.3	0.22	0.32	0.12	0.22	0.17	0.14	0.23	0.22
**Unique reflections**	20662 (1274)*	22424 (1679)	22243 (1685)	18374 (1096)	19183 (1025)	21457 (1415)	24079 (1761)	24074 (1671)	22667 (1450)	19868 (1522)	19332 (1289)	20946 (1509)
**Wavelength (Å)**	1	1	1	1	1	1	1	1	1	1	1	1
**Resolution (Å)**	41.56 - 2.00 (2.11 - 2.00)	37.34 - 2.00 (2.09 - 2.00)	37.00 - 2.00 (2.09 - 2.00)	42.71 - 2.00 (2.11 - 2.00)	43.29 - 2.12 (2.23 - 2.12)	40.02 - 2.00 (2.09 - 2.00)	49.84 - 1.95 (2.03 - 1.95)	43.27 - 1.95 (2.03 - 1.95)	21.34 - 1.95 (2.04 - 1.95)	39.82 - 2.00 (2.11 - 2.00)	43.25 - 2.03 (2.13 - 2.03)	42.68 - 2.00 (2.09 - 2.00)
R_{meas}	0.53 (0.96)	0.29 (3.92)	0.27 (1.64)	0.37 (0.80)	0.25 (2.22)	0.28 (0.86)	0.14 (0.36)	0.16 (1.21)	0.19 (0.71)	0.15 (0.52)	0.20 (0.87)	0.23 (1.05)
$\langle I/\sigma(I)\rangle$	1.97 (1.02)	8.23 (0.85)	7.61 (1.68)	7.79 (2.61)	6.17 (0.44)	7.62 (1.30)	14.46 (6.17)	9.36 (1.08)	8.74 (3.17)	13.50 (5.01)	8.98 (0.99)	7.74 (1.33)
**Completeness (%)**	91.6 (76.0)	99.4 (100)	98.2 (99.8)	81.4 (65.4)	97.1 (71.4)	95.1 (84.4)	99 (99)	98.9 (93.2)	93.2 (80.9)	91.7 (94.8)	92.6 (83.9)	97.6 (94.0)
**Multiplicity**	6.64 (2.36)	12.02 (12.29)	11.62 (11.80)	7.15 (1.81)	8.91 (3.19)	8.43 (3.45)	8.14 (7.93)	4.65 (3.37)	8.42 (8.09)	12.28 (12.01)	8.92 (2.57)	10.30 (5.50)
$CC_{1/2}$	0.95 (0.52)	0.99 (0.22)	0.99 (0.57)	0.95 (0.80)	0.98 (0.33)	0.99 (0.65)	0.99 (0.96)	0.99 (0.45)	0.99 (0.82)	0.99 (0.92)	0.99 (0.60)	0.99 (0.59)
**Wilson B-factor**	22.21	39.11	29.18	29.47	41.7	27.45	15.64	35.73	23.32	24.79	36.86	31.11
**Dose per crystal (kGy)**	213	173	144	58	163	132	58.5	135	50.1	93.4	124	95
**Refinement**												
**Reflections used in refine**	20802 (2438)	22944 (2839)	22104 (2803)	18355 (2107)	19171 (2601)	21512 (2462)	23964 (2644)	24529 (2523)	22529 (2422)	20527 (3016)	20483(2638)	21601 (2570)
**Reflections used for** R_{free}	1041 (122)	1148 (142)	1105 (140)	916 (105)	959 (130)	1075 (123)	1199 (132)	1227 (126)	1127 (121)	1027 (151)	1024 (132)	1081 (129)
R_{work}/R_{free}	0.22/0.26	0.17/0.19	0.19/0.22	0.21/0.26	0.18/0.23	0.20/0.25	0.15/0.19	0.16/0.19	0.18/0.22	0.17/0.21	0.17/0.21	0.19/0.24
**No. of atoms**	2792	2629	2859	2814	2609	2897	3035	2621	2991	2933	2608	2916
**Protein**	2379	2379	2380	2376	2376	2377	2376	2376	2376	2379	2379	2379
**Ligand**	12	12	12	8	8	8	14	14	14	9	9	9
**Solvent**	323	178	383	325	171	401	552	180	496	455	172	423
B **factors (Å^2)**												
**Protein**	22.78	37.94	28.02	30.01	39.64	25.85	11.83	36.42	24.22	21.71	36.66	30.1
**Ligand**	41.79	58.06	43.35	43.00	53.77	40.79	18.40	54.20	25.51	27.79	55.49	31.57
**Solvent**	34.68	67.22	44.07	43.85	63.02	40.94	33.31	63.61	42.97	39.26	62.21	46.39
**R.m.s. deviations**												
**Bond lengths (Å)**	0.012	0.011	0.012	0.015	0.013	0.014	0.013	0.012	0.014	0.013	0.011	0.012
**Bond angles (°)**	1.48	1.37	1.45	1.5	1.44	1.52	1.42	1.36	1.45	1.46	1.42	1.47
**Ramachandran plot**												
**Favoured (%)**	98.48	99.09	98.78	99.09	99.39	98.48	99.09	99.39	98.78	99.09	99.39	99.09
**Allowed (%)**	1.52	0.91	1.22	0.91	0.61	1.52	0.91	0.61	1.22	0.91	0.61	0.91
**Outliers (%)**	0	0	0	0	0	0	0	0	0	0	0	0
**MolProbity clashscore**	1.84	1.66	1.83	3.42	2.29	3.6	2.84	0.83	3.22	4.26	1.67	3.01

*Statistics for the highest-resolution shell are shown in parentheses. **Data processing statistics are reported with Friedel pairs merged.

Table S3. Statistic of the EP-fragment cryoloop-Paratone-N (PN) structures

	TL00150-PN-cryo1	TL00150-PN-RT2	TL00150-PN-cryo3	AC40075-PN-cryo1	AC40075-PN-RT2	AC40075-PN-cryo3
**PDB code**	7H5M	7H5N	7H5L	7H5D	7H5E	7H5C
**Data collection****						
**Temperature (K)**	100	296	100	100	296	100
**Sample delivery**	Cryoloop/Paratone-N	Cryoloop/Paratone-N	Cryoloop/Paratone-N	Cryoloop/Paratone-N	Cryoloop/Paratone-N	Cryoloop/Paratone-N
**No. of crystals**	1	1	1	1	1	1
**Total rotation range (°)**	360	360	360	360	360	360
**Space group**	$P2_1$	$P2_1$	$P2_1$	$P2_1$	$P2_1$	$P2_1$
**Unit-cell parameters**						
a, b, c **(Å)**	44.85, 72.80, 52.53	45.96, 73.98, 53.18	44.94, 72.59, 52.40	43.78, 72.50, 50.82	45.94, 74.02, 52.98	43.36, 71.98, 50.85
**β (°)**	109.16	109.44	109.02	107.55	109.1	107.36
**Mosaicity (°)**	0.39	0.18	0.15	0.15	0.17	0.17
**Unique reflections**	26928 (4242)*	28287 (4521)	26886 (4287)	23672 (3810)	25946 (4099)	23311 (3700)
**Wavelength (Å)**	1	1	1	1	1	1
**Resolution (Å)**	42.36 - 1.86 (1.92 - 1.85)	43.34 - 1.86 (1.92 - 1.86)	42.49 - 1.86 (1.92 - 1.86)	48.45 - 1.91 (1.99 - 1.91)	43.42 - 1.91 (1.99 - 1.91)	20.74 - 1.91 (1.99 - 1.91)
R_{meas}	0.19 (1.28)	0.21 (1.86)	0.10 (0.38)	0.04 (0.11)	0.28 (2.09)	0.09 (0.31)
$\langle I/\sigma(I)\rangle$	7.24 (1.52)	5.93 (1.00)	15.60 (5.39)	35.65 (16.28)	7.51 (0.77)	15.7 (6.14)
**Completeness (%)**	98.9 (96.9)	99.5 (99.2)	99.4 (98.7)	99.7 (99.3)	99.4 (97.7)	99.5 (98.8)
**Multiplicity**	6.88 (6.91)	6.89 (6.8)	6.89 (6.83)	6.97 (7.08)	6.84 (6.98)	6.97 (7.11)
$CC_{1/2}$	0.99 (0.61)	0.99 (0.44)	0.99 (0.95)	0.99 (0.99)	0.99 (0.50)	0.99 (0.97)
**Wilson B-factor**	31.81	35.78	23.13	19.26	41.38	22.58
**Dose per crystal (kGy)**	441	441	441	225	225	225
**Refinement**						
**Reflections used in refine**	26925 (2594)	28280 (2795)	26859 (2651)	23667 (2939)	25934 (2833)	23290 (2862)
**Reflections used for** R_{free}	1347 (130)	1415 (140)	1343 (132)	1184 (147)	1296 (142)	1165 (143)
R_{work}/R_{free}	0.18/0.23	0.21/0.26	0.17/0.20	0.15/0.19	0.18/0.20	0.15/0.19
**No. of atoms**	2878	2651	2874	2912	2612	2937
**Protein**	2382	2379	2382	2381	2379	2379
**Ligand**	12	12	12	9	-	9
**Solvent**	343	176	339	378	164	381
B **factors (Å^2)**						
**Protein**	26.3	32.83	16.53	12.61	40.77	15.36
**Ligand**	42.61	52.90	33.83	19.02	-	19.96
**Solvent**	52.57	64.83	43.78	38.23	71.25	39.97
**R.m.s. deviations**						
**Bond lengths (Å)**	0.013	0.013	0.013	0.016	0.012	0.017
**Bond angles (°)**	1.42	1.42	1.43	1.5	1.39	1.51
**Ramachandran plot**						
**Favoured (%)**	98.48	99.39	99.09	98.78	99.39	98.48
**Allowed (%)**	1.52	0.61	0.91	1.22	0.61	1.52
**Outliers (%)**	0	0	0	0	0	0
**MolProbity clashscore**	2.35	1.63	3.32	5.08	0.78	1.55

*Statistics for the highest-resolution shell are shown in parentheses. **Data processing statistics are reported with Friedel pairs merged.

Table S4. Statistic of the EP-fragment cryoloop-Paraffin (PF) structures

	TL00150-PF-cryo1	TL00150-PF-RT2	TL00150-PF-cryo3	AC39729-PF-cryo1	AC39729-PF-RT2	AC39729-PF-cryo3	JFD03909-PF-cryo1	JFD03909-PF-RT2	JFD03909-PF-cryo3	AC40075-PF-cryo1	AC40075-PF-RT2	AC40075-PF-cryo3
**PDB code**	7H5P	7H5Q	7H5O	7H57	7H58	7H56	7H5V	7H5W	7H5U	7H5G	7H5H	7H5F
**Data collection****												
**Temperature (K)**	100	296	100	100	296	100	100	296	100	100	296	100
**Sample delivery**	Cryoloop/Paraffin	Cryoloop/Paraffin	Cryoloop/Paraffin	Cryoloop/Paraffin	Cryoloop/Paraffin	Cryoloop/Paraffin	Cryoloop/Paraffin	Cryoloop/Paraffin	Cryoloop/Paraffin	Cryoloop/Paraffin	Cryoloop/Paraffin	Cryoloop/Paraffin
**No. of crystals**	1	1	1	1	1	1	1	1	1	1	1	1
**Total rotation range (°)**	360	360	360	360	360	360	360	360	360	360	360	360
**Space group**	$P2_1$	$P2_1$	$P2_1$	$P2_1$	$P2_1$	$P2_1$	$P2_1$	$P2_1$	$P2_1$	$P2_1$	$P2_1$	$P2_1$
**Unit-cell parameters**												
a, b, c **(Å)**	45.39 73.40 52.90	45.93 73.98 53.40	45.16 72.65 52.06	45.33 73.19 52.74	45.18 73.48 51.99	43.63 72.28 50.88	45.23 73.26 52.66	45.93 73.93 53.36	45.19 72.62 52.09	45.27 73.20 52.76	45.80 73.84 52.96	45.01 72.56 51.88
**β (°)**	109.8	109.5	108.92	109.81	108.84	107.67	109.53	109.54	108.94	109.61	109.23	108.72
**Mosaicity (°)**	0.11	0.11	0.17	0.18	0.14	0.08	0.14	0.11	0.15	0.14	0.15	0.15
**Unique reflections**	22548 (3586)*	23124 (3685)	12705 (2028)	22349 (3575)	22432 (3512)	20777 (3316)	22338 (3541)	23129 (3564)	21848 (3496)	26066 (4097)	26685 (4229)	25244 (4019)
**Wavelength (Å)**	1	1	1	1	1	1	1	1	1	1	1	1
**Resolution (Å)**	42.7 - 1.99 (2.08 - 1.99)	43.30 - 1.99 (2.08 - 1.99)	49.25 - 2.39 (2.57 - 2.39)	39.6 - 1.99 (2.08 - 1.99)	40.95 - 1.979 (2.07 - 1.98)	48.48 - 1.99 (2.09 - 1.99)	42.63 - 1.99 (2.08 - 1.99)	43.28 - 1.98 (2.07 - 1.98)	49.26 - 1.99 (2.08 - 1.99)	42.64 - 1.89 (1.96 - 1.89)	43.25 - 1.89 (1.96 - 1.89)	42.62 - 1.89 (1.97 - 1.89)
R_{meas}	0.13 (0.53)	0.19 (1.32)	0.36 (2.91)	0.04 (0.11)	0.15 (2.04)	0.09 (0.46)	0.10 (0.33)	0.16 (1.36)	0.15 (1.13)	0.07 (0.21)	0.15 (1.40)	0.10 (0.71)
$\langle I/\sigma(I)\rangle$	12.15 (3.57)	7.35 (1.11)	4.82 (0.44)	34.42 (15.81)	7.44 (0.90)	14.83 (3.88)	15.75 (5.51)	8.18 (1.14)	9.51 (1.46)	20.09 (8.43)	8.57 (1.14)	13.23 (2.46)
**Completeness (%)**	99.4 (97.4)	99.7 (99.6)	99.8 (99.4)	99.7 (98.9)	99.3 (97.2)	99.7 (99.0)	99.0 (96.8)	99 (95.8)	99.6 (99.3)	99.5 (98.0)	99.6 (98.5)	99.7 (99.4)
**Multiplicity**	6.93 (6.99)	6.96 (7.07)	6.96 (6.59)	6.97 (7.06)	6.88 (7.05)	6.97 (7.12)	6.97 (7.15)	7.00 (7.21)	7.01 (7.15)	6.97 (7.08)	6.96 (7.06)	6.98 (7.09)
$CC_{1/2}$	0.96 (0.92)	0.99 (0.62)	0.99 (0.21)	0.99 (0.99)	0.99 (0.59)	0.99 (0.92)	0.99 (0.96)	0.99 (0.72)	0.99 (0.65)	0.99 (0.98)	0.99 (0.62)	0.99 (0.86)
**Wilson B-factor**	25.51	38.86	51.96	27.98	45.11	36.01	22.09	38.64	35.46	21.2	38.73	32.66
**Dose per crystal (kGy)**	351	351	351	351	351	351	351	351	351	351	351	351
**Refinement**												
**Reflections used in refine**	22539 (2725)	23115 (2860)	12699 (2523)	22347 (2753)	22385 (2694)	20774 (2917)	22332 (2694)	23118 (2744)	21846 (2712)	26061 (2835)	26672 (2595)	25236 (2772)
**Reflections used for** R_{free}	1127 (137)	1156 (143)	636 (127)	1117 (138)	1118 (135)	1039 (146)	1116 (134)	1157 (137)	1093 (136)	1303 (142)	1334 (129)	1263 (139)
R_{work}/R_{free}	0.17/0.22	0.17/0.21	0.21/0.26	0.15/0.20	0.23/0.27	0.18/0.20	0.18/0.23	0.20/0.22	0.21/0.23	0.16/0.19	0.19/0.23	0.19/0.22
**No. of atoms**	2942	2668	2659	2978	2534	2765	2920	2662	2789	2971	2646	2753
**Protein**	2383	2379	2382	2376	2376	2376	2376	2381	2383	2379	2379	2379
**Ligand**	12	-	-	8	-	-	14	-	-	9	-	-
**Solvent**	430	178	176	474	104	242	401	185	253	466	186	263
B **factors (Å²)**												
**Protein**	19.2	37.24	58.75	13.72	53.38	26.64	16.21	37.7	33.72	14.8	36.69	28.62
**Ligand**	32.41	-	-	28.46	-	-	20.06	-	-	22.50	-	-
**Solvent**	41.25	78.54	77.20	39.02	84.00	56.60	38.86	69.05	61.94	38.48	67.31	53.80
**R.m.s. deviations**												
**Bond lengths (Å)**	0.012	0.012	0.012	0.012	0.012	0.013	0.013	0.012	0.013	0.013	0.012	0.013
**Bond angles (°)**	1.44	1.36	1.42	1.38	1.4	1.41	1.42	1.39	1.41	1.4	1.37	1.41
**Ramachandran plot**												
**Favoured (%)**	98.78	99.09	98.48	99.09	99.39	98.78	99.09	98.78	98.48	99.39	99.39	98.78
**Allowed (%)**	1.22	0.91	1.52	0.91	0.61	1.22	0.91	1.22	1.52	0.61	0.61	1.22
**Outliers (%)**	0	0	0	0	0	0	0	0	0	0	0	0
**MolProbity clashscore**	3.38	2.4	2.61	3.39	1.04	3.92	4.15	1.82	3.31	4.38	1.43	5.61

*Statistics for the highest-resolution shell are shown in parentheses. **Data processing statistics are reported with Friedel pairs merged.

Table S5. Statistic of the Thau-Paraffin (PF) and 3CL-Paratone-N (PN) structures

	Thau-PF-cryo	Thau-PF-RT	3CL-PN-cryo	3CL-PN-RT
**PDB code**	9FX4	9FX5	9FX6	9FX7
**Data collection****				
**Temperature (K)**	100	296	100	294
**Sample delivery**	Cryoloop/Paraffin	Cryoloop/Paraffin	Cryoloop/Paratone-N	Cryoloop/Paratone-N
**No. of crystals**	1	1	1	1
**Total rotation range (°)**	360	360	180	140
**Space group**	$P4_12_12$	$P4_12_12$	$P2_12_12_1$	$P2_12_12_1$
**Unit-cell parameters**				
***a, b, c* (Å)**	57.86 57.86 149.90	58.45 58.45 151.50	68.12 99.27 103.67	68.64 101.56 105.26
**β (°)**	90	90	90	90
**Mosaicity (°)**	0.17	0.13	0.19	0.1
**Unique reflections**	35191 (5547)*	36150 (5700)	34960 (5537)	33990 (5399)
**Wavelength (Å)**	1	1	0.97951	0.97951
**Resolution (Å)**	45.81 - 1.59 (1.69-1.59)	46.28 - 1.59 (1.69-1.59)	71.71 - 2.23 (2.36-2.23)	73.09 - 2.28 (2.42-2.28)
R_{meas}	0.09 (2.21)	0.07 (2.31)	0.17 (1.43)	0.14 (1.13)
$\langle I/\sigma(I)\rangle$	23.32 (1.31)	27.46 (1.15)	8.35 (1.45)	7.91 (1.44)
**Completeness (%)**	100 (99.8)	100 (100)	99.6 (99.1)	99.4 (99.4)
**Multiplicity**	25.66(26.39)	26.15 (26.67)	6.58 (6.54)	5.20 (5.39)
$CC_{1/2}$	1.00 (0.86)	1.00 (0.74)	0.99 (0.55)	0.99 (0.63)
**Wilson B-factor**	33.45	34.05	43.44	49.35
**Dose per crystal (kGy)**	351	351	50	77
**Refinement**				
**Reflections used in refine**	35116 (2619)	35962 (2541)	34889 (1528)	33920 (1515)
**Reflections used for** R_{free}	5 (131)	1797 (126)	1660 (90)	1603 (86)
R_{work}/R_{free}	0.18/0.20	0.17/0.18	0.21/0.26	0.19/0.24
**No. of atoms**	1923	1830	5104	4959
**Protein**	1602	1601	4700	4705
**Ligand**	-	-	-	-
**Solvent**	311	219	373	223
***B* factors (Å^2)**				
**Protein**	31.46	30.78	41.26	49.36
**Ligand**	-	-	-	-
**Solvent**	45.38	48.14	50.75	65.76
**R.m.s. deviations**				
**Bond lengths (Å)**	0.013	0.013	0.011	0.011
**Bond angles (°)**	1.46	1.42	1.42	1.39
**Ramachandran plot**				
**Favoured (%)**	98.05	98.05	97.97	96.96
**Allowed (%)**	1.95	1.95	2.03	3.04
**Outliers (%)**	0	0	0	0
**MolProbity clashscore**	1.9	1.9	6.1	6.2

*Statistics for the highest-resolution shell are shown in parentheses. **Data processing statistics are reported with Friedel pairs merged.

Table S6. Occupancy and B-factor from Buster refinement for the TL00150, AC39729, JFD03909, and AC40075 at both cryo and RT

Sample	Sample setup	cryo1 occupancy/B-factor	RT2 occupancy/B-factor	cryo3 occupancy/B-factor
**TL00150**	IMISX chip	0.83/42	0.71/58	0.84/43
**AC39729**	IMISX chip	0.94/43	0.87/54	0.99/41
**JFD03909**	IMISX chip	0.89/18	0.68/54	0.84/26
**AC40075**	IMISX chip	0.91/28	0.65/55	0.81/32
**TL00150**	cryoloop (Paratone-N)	0.76/43	0.59/53	0.74/34
**AC40075**	cryoloop (Paratone-N)	0.84/19	-	0.71/20

Table S7. Structure comparison of the EP structures in complex with fragments at cryo and RT conditions

Sample	Sample setup	r.m.s.d.<br>RT2 v.s. cryo1	r.m.s.d.<br>cryo3 v.s. cryo1
**TL00150**	IMISX chip	0.263	0.137
**AC39729**	IMISX chip	0.323	0.075
**JFD03909**	IMISX chip	0.280	0.068
**AC40075**	IMISX chip	0.284	0.084
**TL00150**	cryoloop (Paratone-N)	0.291	0.078
**AC40075**	cryoloop (Paratone-N)	0.387	0.102
The alignment was made by aligning the backbone of the EP from residues 1 to 330.<br>r.m.s.d. stands for root mean square deviation.			
